# Longitudinal changes and cumulative exposure of cardiometabolic index predict cardiovascular disease risk in middle-aged and older adults: a national prospective cohort study

**DOI:** 10.1186/s12872-026-06101-3

**Published:** 2026-06-27

**Authors:** Xiaoyun Liu, Lu Yin, Xiaohua Hao

**Affiliations:** 1 Department of Phase I Clinical Trail Center, Beijing Shijitan Hospital, Capital Medical University, No. 10 Tieyi Road, Yangfangdian, Haidian District, Beijing, 100038 China; 2 Fuwai Hospital, National Clinical Research Center for Cardiovascular Diseases (NCRC), Chinese Academy of Medical Sciences and Peking Union Medical College, National Center for Cardiovascular Diseases, Beijing, 100037 China

**Keywords:** Cardiometabolic index, Cardiovascular disease, Longitudinal change, Cumulative exposure, Risk prediction

## Abstract

**Background:**

Cardiovascular disease (CVD) remains the leading cause of morbidity and mortality among aging populations. Single-time-point risk assessments may not reflect dynamic cardiometabolic changes. The cardiometabolic index (CMI), integrating triglyceride-to-HDL cholesterol ratio and waist-to-height ratio, reflects both atherogenic dyslipidemia and central adiposity, but its longitudinal prognostic value remains unclear. Thus, this study aims to evaluate the associations of baseline CMI, longitudinal change patterns, cumulative exposure, and absolute change (ΔCMI) with incident CVD among Chinese adults aged ≥ 45 years.

**Methods:**

Data from the China Health and Retirement Longitudinal Study (CHARLS). Baseline analyses included 9120 participants. Longitudinal analyses included 6164 participants. CVD (including heart diseases and stroke) was ascertained during follow-up. Multivariable Cox proportional hazard models were fitted with adjustment for confounding factors.

**Results:**

Higher baseline CMI was independently associated with increased CVD risk with a non-linear association and an inflection point at 0.42. Compared with stable low CMI, participants with persistently high CMI showed the highest CVD risk (HR = 1.31; 95%CI: 1.14, 1.50), followed by progression from low-to-high CMI (HR = 1.23; 95%CI: 1.04, 1.46), whereas improvement from high-to-low was not associated with excess risk. Among participants with low baseline CMI, higher cumulative CMI and less favorable ΔCMI were associated with increased CVD risk. Incorporating longitudinal CMI changes modestly but significantly improved risk discrimination and reclassification beyond baseline assessment.

**Conclusions:**

CMI is a dynamic, threshold-dependent predictor of CVD, independent of LDL-C and hsCRP. Longitudinal monitoring enhances risk stratification beyond single measurements.

**Supplementary Information:**

The online version contains supplementary material available at https://doi.org/10.1186/s12872-026-06101-3.

## Background

Cardiovascular disease (CVD) remains one of the leading causes of mortality and disability worldwide [1, 2]. Although age-standardized rates have declined in some settings, the overall burden of CVD is expected to remain substantial in the coming decades, with metabolic risk factors contributing an increasing proportion of this burden [3, 4]. Conventional risk assessment has largely relied on single time point measurements of low-density lipoprotein cholesterol (LDL-C), blood pressure, and glycaemia. However, growing evidence suggests that cardiometabolic risk is dynamic and accumulative over the life course. Insulin resistance (IR) and central adiposity are two key mechanisms linking metabolic dysfunction to atherosclerosis, and subsequent cardiovascular events [5, 6]. However, single baseline measurements may therefore provide only limited information on the longitudinal burden of these processes. Therefore, identifying practical markers that reflect both cardiometabolic status and its longitudinal evolution over time may help improve cardiovascular risk stratification.

The triglyceride to high-density lipoprotein cholesterol (TG/HDL-C) ratio has been widely used as a surrogate indicator of IR, and previous studies have reported its association with CVD risk [7, 8]. Moreover, obesity, particularly central obesity, is also emerging as the fastest-growing risk factor for cardiovascular morbidity and mortality worldwide [3]. By 2030, nearly 3 billion adults, approximately half of the global adult population, will be overweight/obesity [9]. Although body mass index (BMI) is commonly used to classify obesity, it does not provide precise information about the body composition or fat distribution, which is closely related to cardiometabolic risk [10]. A large study based on the UK Biobank involving over 500,000 adults indicated that waist-to-height ratio (WHtR) may outperformto BMI and body fat percentage in predicting CVD risk, highlighting the clinical relevance of central adiposity [11].

The cardiometabolic index (CMI), combining TG/HDL-C ratio and WHtR, integrates key components of atherogenic dyslipidaemia and central obesity and has been proposed as a simple marker for cardiometabolic risk [12, 13]. Previous studies have examined TG/HDL-C ratio or WHtR individually as predictors of cardiovascular events, and studies of CMI have also suggested that higher levels are associated with CVD risk [14–17]. Notably, some studies have suggested that, compared with TG/HDL-C ratio alone, CMI may have stronger predictive value for ischemic CVD [14]. This potential advantage lies in that CMI jointly captures lipid-related and central obesity-related abnormalities, rather than reflecting only one dimension of metabolic dysfunction. However, evidence regarding the shape and strength of the CMI-CVD association remains inconsistent, with both linear and nonlinear associations having been reported [18, 19]. Moreover, most prior studies have a assessed CMI at a single time point, with limited attention to how long-term patterns of CMI may be associated with future CVD risk. Because CMI jointly captures lipid-related and central obesity related abnormalities, a longitudinal evaluation of CMI may provide a more integrated characterization of cumulative cardiometabolic burden than either component alone. To our knowledge, few population-based studies have comprehensively evaluated baseline CMI together with multiple longitudinal CMI metrices in relation to CVD risk.

Therefore, using data from the China Health and Retirement Longitudinal Study (CHARLS), the present study first aimed to examined the association between baseline CMI and CVD risk. Thenwe utilized long-term follow-up data to conduct a comprehensive evaluation of CMI dynamics, including changes patterns, cumulative exposure and absolute changes, to assess their associations with future CVD risk.

## Materials and methods

The CHARLS is an ongoing national longitudinal survey that aims to be representative of the residents in mainland China aged 45 and older. It employs a multistage stratified probability-proportional-to-size sampling strategy to ensure sample representativeness. Detailed information on the study design and sampling procedures have been published previously [20]. In the present study, we used data from four survey waves conducted between 2011 and 2018 (Wave 1: 2011–2012; Wave 2: 2013; Wave 3: 2015; Wave 4: 2018; data are available at http://charls.pku.edu.cn/en). The CHARLS was approval by the Institutional Review Board at Peking University, and written informed consent forms were obtained from each participant.

### Study participants

**Fig. 1:**
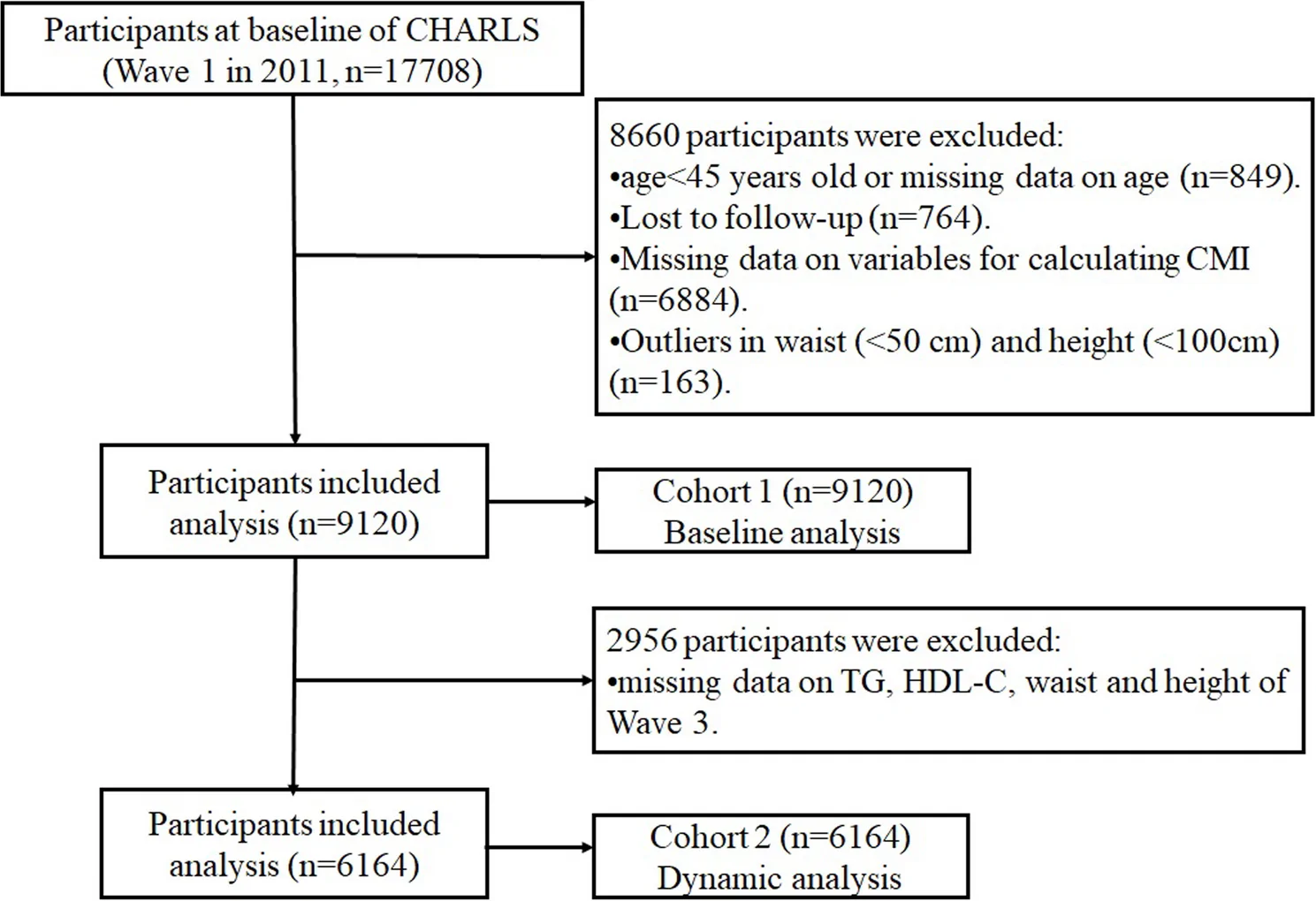
The flowchart of the study participants

The baseline survey (Wave 1) involved 17708 individuals in 10257 households across 150 countries/districts, 450 villages/urban communities, within 25 provinces throughout China. Subsequent follow-up surveys were conducted every 2 years with standardized questionnaire interviews and anthropometric measurements. For the present study, participants were excluded if they were younger than 45 years or had missing data on age, lacked follow-up date, had missing baseline data required for calculating CMI (TG, HDL-C, waist and height), or had outliers in anthropometric measurements (Fig. 1). The remaining 9120 participants were included in baseline CMI analysis (Cohort 1). For analyses of longitudinal changes in CMI, we further excluded participants without complete CMI-related measurements at Wave 3. The final sample for dynamic CMI analyses comprised 6164 participants (Cohort 2).

### Assessment of cardiometabolic index

CMI was calculated at baseline and at Wave 3 using the following formula:

$$\mathrm{CMI}=\mathrm{TG}\;\left(\mathrm{mmol}/{\mathrm{L}}\right)/{\mathrm{HDL-C}}\;\left(\mathrm{mmol}/{\mathrm{L}}\right)*\mathrm{waist}\left(\mathrm{cm}\right)/{\mathrm{height}}\left(\mathrm{cm}\right)\left(12\right)$$

The exposures of interest included baseline CMI, change in CMI patterns, cumulative CMI exposure and the absolute change in CMI (ΔCMI). Baseline CMI were categorized into quantiles: Q1 < 0.29 (reference group), 0.29 *≤* Q2 < 0.48, 0.48 *≤* Q3 < 0.85, Q4 *≥* 0.85. According to the results of restricted cubic-spline analysis, high CMI level were defined as *≥* 0.42. For longitudinal analyses Cohort 2, participants were classified into four groups according to CMI levels at baseline and Wave 3, defined as changes in CMI patterns. Those with persistently low CMI levels were placed in the “stable low” group, and those with persistently high CMI levels were in the “stable high” group. Those who transitioned from low to high levels of CMI were assigned to the “low to high” group, and those who transitioned from high to low levels were in the “high to low” group. Cumulative CMI exposure were calculated as the time-weighted average of CMI between 2011 and 2015 using the following formula: (CMI_2011_ + CMI_2015_) / 2 * time (2015 − 2011). We calculated the ΔCMI with the formula: CMI_2015_ - CMI_2011_.

### Outcome ascertainment

The primary outcome was CVD, defined as the composite of self-reported physician diagnoses heart diseases or stroke. The secondary outcomes were the components of the primary outcome, including heart diseases and stroke. Detailed information on outcome ascertainment and validation were showed in Supplementary Methods. For baseline CMI analyses, follow-up was initiated from 2011, with subsequent follow-up conducted every 2 years (2013 to 2018). For dynamic change analyses, follow-up was initiated from 2015 (Wave 3), with subsequent follow-up at 2018 (Wave 4). For all events we considered the first occurrence of the event interest.

### Covariates

 Baseline covariates included age, sex, education level, smoking habit, drinking status, LDL-C, glucose, systolic blood pressure (SBP), diastolic blood pressure (DBP), high-sensitivity C-reactive protein (hsCRP) and medication use (including antihypertension and lipid-lowering medication). Additional details are provided in the Supplementary Methods section [20, 21]. Hypertension was defined as SBP *≥* 140 mmHg and/or DBP *≥* 90 mmHg, current use of antihypertension medication, or self-reported hypertension [22].

### Statistical analysis

For descriptive statistics, continuous variables were expressed as mean [standard deviation (SD)], while categorical variables were expressed as number (percentage).

For baseline CMI anslysis, Cox proportional hazard regression was used to calculate the hazard ratios (HRs) and 95% confidence intervals (CIs). Three models were constructed: Model 1 was unadjusted; Model 2 adjusted for age and sex; Model 3 additionally adjusted for smoking habit, drinking, education level, LDL-C, glucose, SBP, DBP, hsCRP and lipid-lowering medication. Linear trend tests across quantiles were performed by modeling quartiles as a continuous variable. Restricted cubic-spline plots with three knots (the 10th, 50th and 90th percentiles) were used to explore the shape of the association between baseline CMI and CVD. If non-linearity was observed, the maximally selected rank statistics was applied to determine an optimal cut-off in Cohort 1; the cut-off was then applied in Cohort 2 to avoid overfitting.

For longitudinal analyses (change in CMI patterns, cumulative CMI and ΔCMI), two models were fitted: Model 1 were adjusted for age, sex, smoking habit, drinking, education level, LDL-C, glucose, SBP, DBP, hsCRP and lipid-lowering medication; Model 2 were additionally adjusted for baseline CMI levels. The missing covariate data were imputed using the multiple imputation with chained equation (Supplementary Methods and Table S1) [23].

Several sensitivity analyses were conducted using model 3 to test robustness: (i) analyses of heart diseases and stroke separately; (ii) complete-case analysis without imputation; (iii) inverse probability weighting (IPW); (iv) subgroups analyses stratified by hypertension status, sex, and hsCRP categories (< 1 mg/L, 1–3 mg/L and *≥* 3 mg/L). To explore potential reasons of CMI reduction, changes of modifiable factors during follow-up were compared between participants with stable high CMI and participants with high baseline CMI who recovered to low CMI.

To assess the incremental predictive value of changes in CMI, we constructed ROC curves for predicting 3-year CVD risk, and compared models with and without changes in CMI using C-statistic, integrated discrimination improvement (IDI), and category-based/continuous net reclassification improvement (NRI). Category-based NRI was calculated using predefined risk categories (< 5%, 5–10%, and > 10%). Model calibration was assessed by estimating calibration slopes. Graphical calibration was further evaluated using decile-based calibration plots, in which participants were grouped according to predicted risk and observed risks were estimated using the Kaplan-Meier method.

All analysis were performed using R statistical software (version 4.4.2). We considered two-sided P value < 0.05 to be significant.

## Results

### Participant characteristics

For baseline CMI analyses (Cohort 1), characteristics of the 9120 participants according to quartiles of CMI are presented in Table S2. The mean baseline CMI was 0.80 *±* 1.41, the mean age was 59.3 years, and 53.3% were female. During a median follow-up of 8 years, only the first occurrence of the outcome was considered per participant.

For analyses of longitudinal changes in CMI (Cohort 2), 6164 participants were included (Table 1). The mean age was 58.8 years and 54.0% were female, with a median follow-up duration of 4 years. Compared with participants with stable low CMI, those in other groups were more likely to be female and less likely to be current smokers or alcohol consumers. They also exhibited a less favorable cardiometabolic profile, including higher BMI, waist, SBP, DBP, TC, TG, LDL-C, glucose, and hsCRP levels, as well as lower HDL-C levels. Figure S1 shows the trends of CMI and its component levels from baseline to Wave 3. Compared with baseline levels, CMI decreased (*p* < 0.0001), while both HDL-C and TG levels increased at Wave 3 (both *p* < 0.01).

**Table 1:** Baseline characteristics of 6164 participants according to changes in CMI

	Overall (*n* = 6164)	Changes in CMI				*p*-value^*^
		Stable low (*n* = 1973)	Low to high (*n* = 1004)	High to low (*n* = 645)	Stable high (*n* = 2542)	
Age, years	58.8 (8.7)	59.5 (8.9)	58.3 (8.8)	58.7 (8.5)	58.4 (8.5)	< 0.001
Sex						
male	2837 (46.0)	1077 (54.6)	439 (43.7)	312 (48.4)	1009 (39.7)	< 0.001
female	3327 (54.0)	896 (45.4)	565 (56.3)	333 (51.6)	1533 (60.3)	
BMI, *kg/m*^*2*^	23.6 (3.9)	21.6 (3.2)	23.4 (3.4)	23.2 (3.1)	25.4 (3.8)	< 0.001
Waist, *cm*	85.6 (10.0)	79.8 (8.1)	84.5 (8.8)	84.5 (8.6)	90.7 (9.4)	< 0.001
Height, *cm*	157.9 (8.3)	158.2 (8.2)	157.6 (8.5)	158.0 (8.3)	157.9 (8.4)	0.378
SBP, *mmHg*	129.1 (21.1)	125.3 (20.4)	127.2 (20.1)	130.3 (21.1)	132.4 (21.3)	< 0.001
DBP, *mmHg*	75.3 (12.1)	72.6 (11.7)	74.6 (11.4)	75.4 (11.9)	77.5 (12.3)	< 0.001
TC, *mmol/L*	5.0 (1.0)	4.9 (0.9)	5.0 (1.0)	5.0 (1.0)	5.2 (1.1)	< 0.001
TG, *mmol/L*	1.5 (1.2)	0.8 (0.3)	0.9 (0.2)	1.8 (1.3)	2.2 (1.5)	< 0.001
HDL-C, *mmol/L*	1.3 (0.4)	1.6 (0.4)	1.5 (0.3)	1.2 (0.2)	1.1 (0.3)	< 0.001
LDL-C, *mmol/L*	3.0 (0.9)	2.9 (0.8)	3.2 (0.9)	2.9 (0.9)	3.1 (1.0)	< 0.001
Glucose, *mmol/L*	6.1 (2.0)	5.8 (1.4)	5.8 (1.4)	6.2 (2.0)	6.5 (2.4)	< 0.001
WBC *in thousands*	6.3 (1.9)	6.0 (1.9)	6.1 (1.9)	6.3 (1.9)	6.5 (1.9)	< 0.001
hsCRP, *mg/L*	2.5 (6.8)	2.3 (7.8)	2.8 (7.8)	2.4 (8.3)	2.6 (4.9)	0.211
Smoking habit, n (%)						
never	3777 (61.3)	1077 (54.6)	641 (63.8)	402 (62.3)	1657 (65.2)	< 0.001
former	535 (8.7)	175 (8.9)	73 (7.3)	51 (7.9)	236 (9.3)	
current	1847 (30.0)	720 (36.5)	289 (28.8)	192 (29.8)	646 (25.4)	
Drinking, n (%)						
No	3772 (61.2)	1073 (54.4)	623 (62.1)	391 (60.6)	1685 (66.3)	< 0.001
Yes	2384 (38.7)	897 (45.5)	381 (37.9)	254 (39.4)	852 (33.5)	
Education level, n (%)						
Low	5641 (91.5)	1823 (92.4)	917 (91.3)	598 (92.7)	2303 (90.6)	0.218
Middle	470 (7.6)	133 (6.7)	81 (8.1)	40 (6.2)	216 (8.5)	
High	53 (0.9)	17 (0.9)	6 (0.6)	7 (1.1)	23 (0.9)	
Medication use, n(%)						
Antidiabetic	234 (3.8)	52 (2.6)	25 (2.5)	12 (1.9)	145 (5.7)	< 0.001
Antihypertension	1218 (19.8)	236 (12.0)	159 (15.8)	120 (18.6)	703 (27.7)	< 0.001
Lipid-lowering	341 (5.5)	48 (2.4)	47 (4.7)	13 (2.0)	233 (9.2)	< 0.001
Hypertension, n(%)						
No	3599 (58.4)	1367 (69.3)	628 (62.5)	371 (57.5)	1233 (48.5)	< 0.001
Yes	2545 (41.3)	596 (30.2)	373 (37.2)	273 (42.3)	1303 (51.3)	
Diabetes, n(%)						
No	5123 (83.1)	1761 (89.3)	895 (89.1)	534 (82.8)	1933 (76.0)	< 0.001
Yes	1041 (16.9)	212 (10.7)	109 (10.9)	111 (17.2)	609 (24.0)	
Baseline CMI	0.81 (1.45)	0.27 (0.10)	0.33 (0.09)	0.91 (1.81)	1.40 (1.90)	< 0.001

*BMI* Body mass index, *BP* Blood pressure, *WBC* White blood cell, *TC* Total cholesterol, *TG* Triglyceride, *HDL-C* High-density lipoprotein-cholesterol

Data was expressed as mean (SD) for continuous variables and frequency (percentage) for categorical variables

^*^ *p* values were obtained by ANOVA for continuous variables or the chi-square test for categorical variables

### Associations between baseline CMI and CVD risk

Associations between baseline CMI quartiles and CVD risk are presented in Table S3. In the fully adjusted model, higher baseline CMI was significantly associated with increased risk of CVD. Compared with the lowest quartile, the adjusted HRs were 1.15 (95% CI: 1.03, 1.29) for Q2, 1.26 (95% CI: 1.12, 1.41) for Q3, and 1.33 (95% CI: 1.18, 1.48) for Q4 (*p* for trend < 0.001). Higher CMI was independently associated with an increased risk of CVD after accounting for the competing risk of non-cardiovascular death (Table S31, Figure S5).

**Fig. 2:**
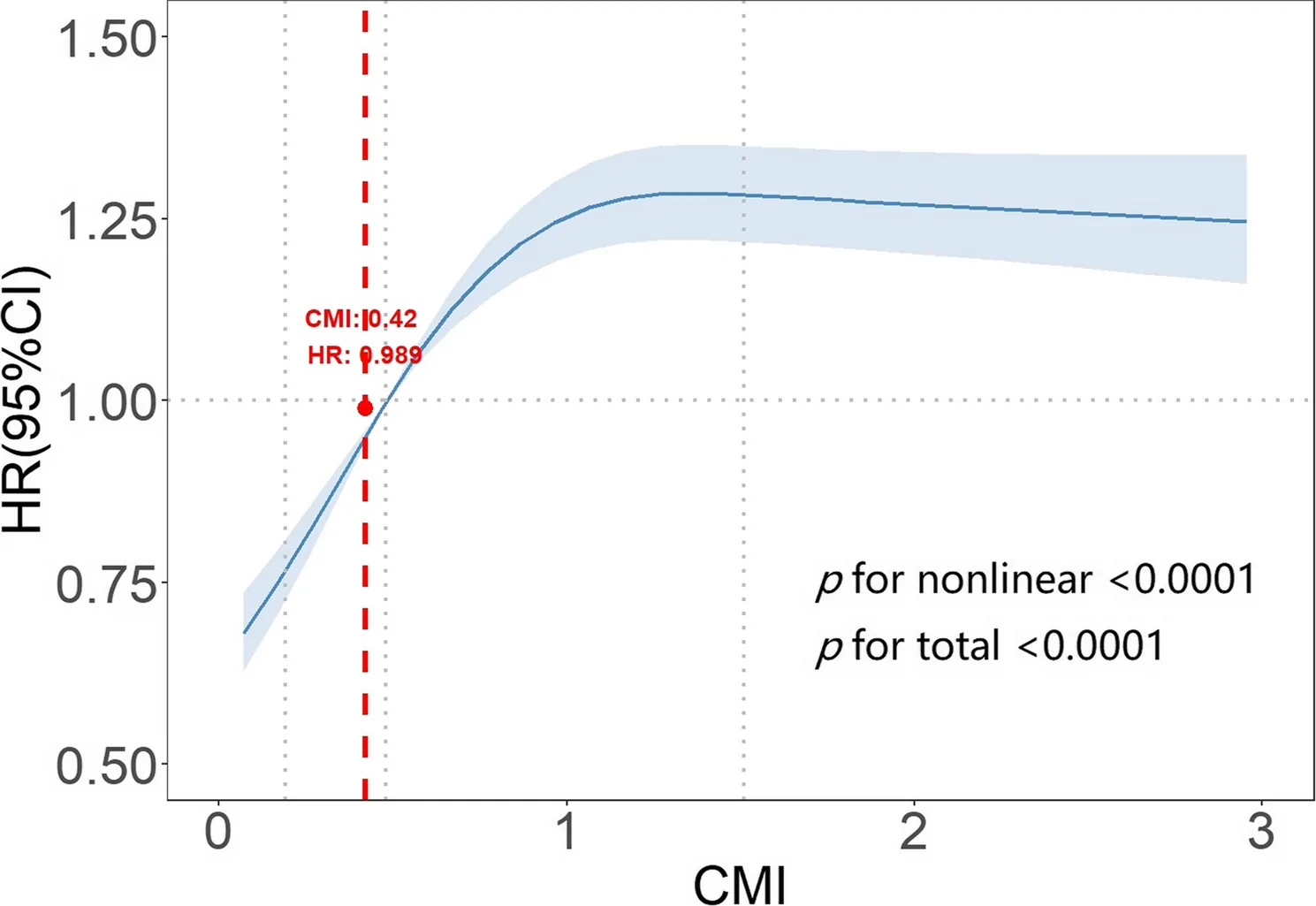
Restricted cubic spline plot associations of baseline CMI with CVD in total participants. The median CMI was reference standard (0.48). The red line represents the threshold value of CMI at 0.42

Restricted cubic spline analysis revealed a significant nonlinear association of baseline CMI and CVD risk (*p* for non-linearity < 0.0001; Fig. 2). Maximally selected rank statistics identified a CMI value of 0.42 as the optimal cut-off for risk discrimination (Figure S2). Based on this threshold, participants were categorized into high-risk (CMI *≥* 0.42) and low-risk (CMI < 0.42) groups for subsequent analyses.

### Associations of longitudinal CMI change patterns with CVD

Associations between changes in CMI patterns and CVD risk are shown in Table 2. After multivariable adjustment, the lowest risk of CVD occurred in participants with stable low CMI. Compared with this reference group, those with stable high CMI showed the highest risk (HR = 1.31; 95% CI: 1.14, 1.50), followed by participants with low baseline CMI who progressed to high CMI (1.23; 1.04, 1.46). In contrast, participants with high baseline CMI who recovered to low did not show a statistically significant excess risk. These associations remained consistent after further adjusting for baseline CMI.

**Table 2:** Associations of changes in CMI with CVD

	Event proportion	Model 1		Model 2	
		HR (95%CI)	*P* value	HR (95%CI)	*P* value
Stable low	323/1632 (19.8)	Ref.		Ref.	
Low to high	241/990 (24.3)	1.23 (1.04, 1.46)	0.015	1.23 (1.04, 1.46)	0.016
High to low	142/629 (22.6)	1.12 (0.92, 1.37)	0.254	1.12 (0.92, 1.37)	0.265
Stable high	836/2913 (28.7)	1.31 (1.14, 1.50)	< 0.001	1.30 (1.13, 1.50)	< 0.001

Model 1 adjusted for age, sex, smoking habit, drinking, education level, LDL-C, glucose, SBP, DBP, hsCRP at baseline and medication use of lipid-lowering. Model 2 additionally adjusted for baseline CMI

### Associations between cumulative CMI exposure and CVD risk

Stratified by baseline CMI risk status, associations between cumulative CMI exposure and CVD are presented in Table 3. Among participants with low-risk baseline CMI (< 0.42), higher cumulative CMI was associated with an increased risk of CVD (*p* for trend = 0.039). Compared with the lowest tertile, adjusted HRs were 1.00 (0.73, 1.36) for tertile 2 and 1.22 (0.89, 1.67) for tertile 3. In contrast, no significant association was observed in participants with high-risk baseline CMI (*p* for trend = 0.683). These findings remained unchanged after further adjustment for baseline CMI.

**Table 3:** Associations of cumulative CMI with CVD

	Event proportion	Model 1		Model 2	
		HR (95%CI)	*P* value	HR (95%CI)	*P* value
Overall (*n* = 6164)					
T1	554/2076 (26.7%)	Ref.		Ref.	
T2	468/2040 (22.9%)	0.91 (0.80, 1.03)	0.119	0.92 (0.81, 1.05)	0.205
T3	520/2048 (25.4%)	0.98 (0.87, 1.11)	0.797	1.00 (0.88, 1.13)	0.966
P for trend		0.782		0.995	
Participants with low baseline CMI (< 0.42) (*n* = 2622)					
T1	48/251 (19.1%)	Ref.		Ref.	
T2	271/1330 (20.4%)	1.00 (0.73, 1.36)	0.998	1.00 (0.73, 1.36)	0.975
T3	245/1041 (23.5%)	1.22 (0.89, 1.67)	0.212	1.22 (0.89, 1.67)	0.225
P for trend		0.039		0.039	
Participants with high baseline CMI (≥ 0.42)) (*n* = 3542					
T1	506/1825 (27.7%)	Ref.		Ref.	
T2	197/710 (27.7%)	1.03 (0.87, 1.22)	0.695	1.04 (0.88, 1.23)	0.666
T3	275/1007 (27.3%)	0.96 (0.83, 1.12)	0.631	0.97 (0.83, 1.13)	0.663
P for trend		0.683		0.712	

Model 1 adjusted for age, sex, smoking habit, drinking, education level, LDL-C, glucose, SBP, DBP, hsCRP at baseline and medication use of lipid-lowering. Model 2 additionally adjusted for baseline CMI

### Associations betweenabsolute change in CMI and CVD risk

Associations between absolute change in (ΔCMI) and CVD are shown in Table S7. Among participants with low-risk baseline CMI, a greater reduction in ΔCMI was associated with lower CVD risk. Compared with the upper tertile, participants in the middle and lowest tertiles had HRs of 0.82 (95% CI: 0.69–0.98) and 0.82 (0.60–1.12), respectively (*p* for trend = 0.027). No significant associations were observed among participants with high-risk baseline CMI. These associations were not attenuated by further adjustment for baseline CMI.

### Subgroup analyses

Subgroup analyses stratified by hypertension status are shown in Table S17-S20. Among participants with hypertension, increasing baseline CMI was associated with progressively higher risk of CVD and stroke. Compared with participants with the combination of stable low CMI and hypertension, those with stable high CMI and those with low-to-high CMI were marginally associated with an increased CVD risk, whereas those whose CMI decreased from high to low did not show a significant excess risk.

Sex-specific analyses demonstrated that associations of baseline CMI, changes in CMI, cumulative CMI and ΔCMI with CVD were generally stronger in males than in females (Table S22-S24). When stratified by hsCRP levels, higher baseline CMI was associated with increased risks of CVD, heart diseases and stroke across all hsCRP levels, with the strongest associations observed among individuals with hsCRP *≥* 3 mg/L (Table S25). Among participants with hsCRP of 1–3 mg/L, both participants with stable high CMI and those with low baseline CMI who progressed to high CMI were significantly associated with increased CVD risk (Table S26). A similar trend was observed in the highest hsCRP group, although confidence intervals were wider.

### Sensitivity analyses

Sensitivity analyses yielded results consistent with the primary findings. Baseline CMI was positively associated with risks of heart diseases and stroke (Table S3). Associations of CMI changes patterns, cumulative CMI, and ΔCMI with heart diseases and stroke were consistent with primary analyses (Tables S4-S6 and S8-S9). Analyses using the complete dataset without imputation produced similar results (Tables S10-S13). Inverse probability weighting reduced baseline differences between included and excluded participants (Table S14 and Figure S3), and weighted analyses confirmed the robustness of the primary findings (Tables S15-S16).

### Modifiable factors associated with CMI reduction

Changes in modifiable risk factors during follow-up associated with CMI reduction are presented in the Supplementary data (Table S29). In univariable analysis, returning to a normal BMI, increasing physical activity and returning to normal sleep duration were associated with a higher likelihood of CMI reduction. However, an increase in hsCRP was more likely to be associated with increased CMI levels. Participants with stable high CMI were more likely to initiate antidiabetic or lipid-lowering therapy during follow-up compared with those who recovered to low levels.

### Incremental predictive value of changes in CMI

**Fig. 3:**
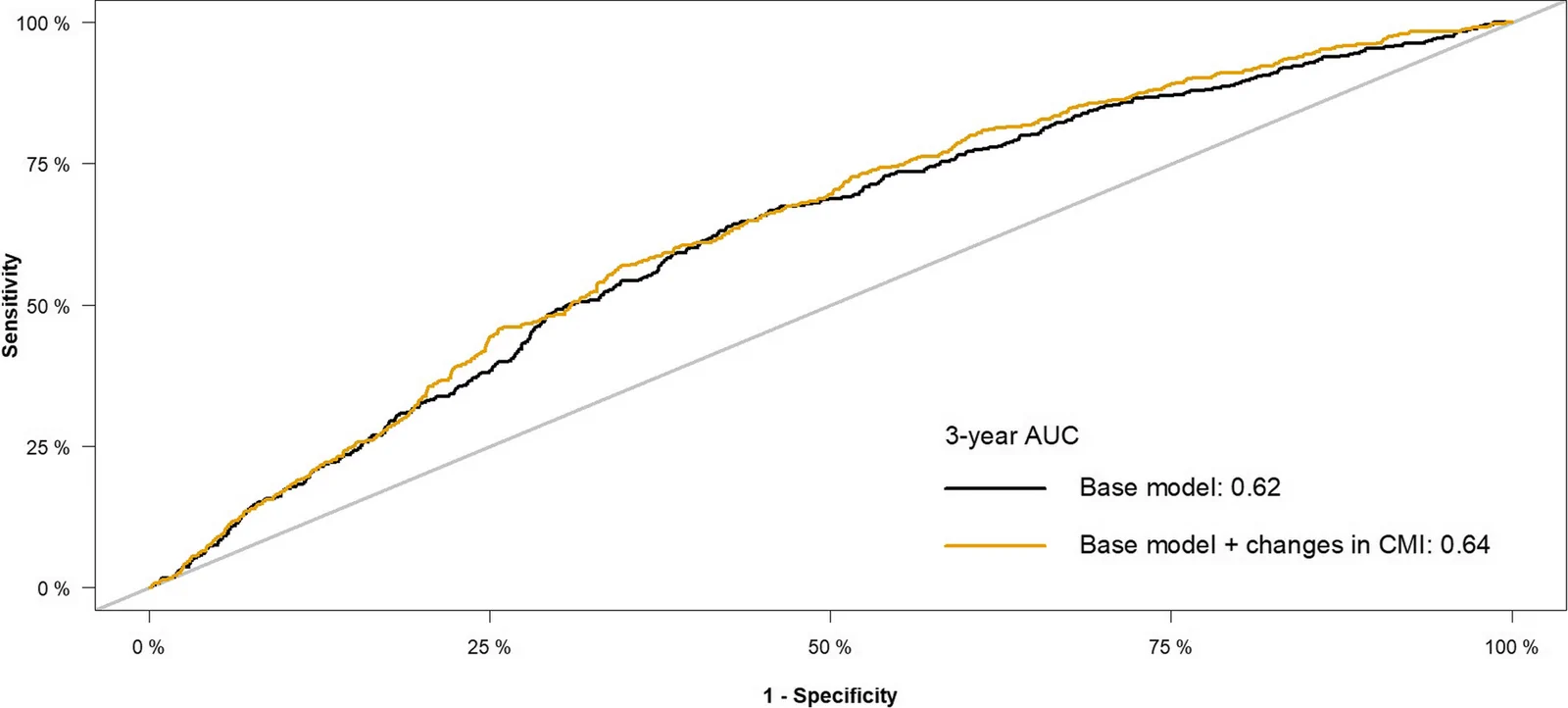
ROC curves for the base model and the base model plus changes in CMI for predicting 3-year CVD risk. Base model includes baseline CMI, age, sex, smoking habit, drinking, education level, LDL-C, glucose, SBP, DBP and medication use of lipid-lowering

We further conducted additional analyses by adding changes in CMI to baseline models (Fig. 3, Table S30). Compared with the baseline model, the extended model that included changes in CMI showed a significant improvement in risk discrimination, with the 3-year AUC increasing from 0.62 (95% CI: 0.60, 0.65) to 0.64 (95% CI: 0.61, 0.66) and with the ΔC-statistic increasing by 0.007 (95% CI: 0.003, 0.012). Although the category-based NRI did not reach statistical significance (0.018; 95% CI: -0.018, 0.051), both continuous NRI (0.274; 95% CI: 0.135, 0.364) and IDI (0.002; 95% CI: 0.0007, 0.0035) demonstrated significant improvements. The calibration slope increased from 0.84 (0.63, 1.05) in the base model to 0.91 (95% CI: 0.70, 1.11) in the extended model. Decile-based calibration plots at 3 years showed good agreement between predicted and observed risks, with no evidence of systematic over- or underestimation across risk strata (Figure S4). Although the AUC increase was modest, the continuous NRI and IDI improved significantly, and calibration remained acceptable, supporting the incremental predictive value of changes in CMI.

## Discussion

In this large national prospective cohort study of Chinese adults aged 45 years and older, we found that baseline CMI was associated with CVD risk in a nonlinearmanner. More importantly, longitudinal CMI change patterns and cumulative CMI exposure were also associated with CVD risk beyond a single baseline assessment. Several key findings were found.

First, higher baseline CMI was independently associated with an increased CVD risk after extensive adjustment, including LDL-C and hsCRP. These findings suggests that CMI may captures aspects of cardiometabolic burden not fully reflected by conventional lipid measures or inflammation alone. Notably, restricted cubic spline analyses revealed a non-linear association between CMI and CVD risk. We identified 0.42 as the optimal cut-off for subsequent stratified analyses within this dataset. However, because this threshold was derived and evaluated in the same cohort, it should be considered exploratory rather than definitive, and external validation is required before any broader clinical or screening application can be considered.

Second, longitudinal changes in CMI provided important prognostic information. Participants who maintained persistently high CMI had the highest CVD risk, and those who progressed from low to high CMI exhibited a comparably elevated risk, suggesting that worsening cardiometabolic status over time was associated with an unfavorable cardiovascular risk profile. In contrast, participants whose CMI improved from high to low did not show a statistically significant excess risk compared with those who remained stable low. Collectively, these findings support that CMI may serve as a dynamic marker of cardiometabolic risk. However, given the observational design, these associations should not be interpreted as evidence that reducing CMI directly lowers CVD risk. In addition, although longitudinal CMI changes were associated with incident CVD, the observed improvements in predictive performance were modest. Therefore, these findings should be interpreted as supporting the epidemiological relevance of long-term CMI exposure rather than establish immediate clinical utility for risk stratification or decision-making.

Third, analyses of cumulative exposure and absolute change highlighted the potential relevance of long-term CMI burden, particularly among participants with among participants with low baseline CMI (< 0.42). In this group, higher cumulative CMI was associated with an increased CVD risk, and greater CMI reductions (more favorable ΔCMI) were associated with lower risk. These findings imply that even when baseline CMI is relatively low, the accumulation of adverse cardiometabolic burden over time may still be relevant to future cardiovascular risk. By contrast, among participants with high baseline CMI (*≥* 0.42), neither cumulative CMI nor ΔCMI was significantly associated with CVD risk. One possible explanation is that cardiometabolic burden in this group was already reached a risk plateau at baseline, such that short-term increases over the subsequent period contribute relatively little additional risk. Alternatively explanations include limited statistical power, residual heterogeneity, and differences in clinical management during follow-up, such as the initiation of antidiabetic or lipid-lowering therapy.Notably, our analyses of dynamic changes also found that improvement from high to low CMI was not associated with excess risk; however, this does not prove that a protective effect of CMI reduction.

CMI was first proposed by Wakabayashi and colleagues in 2015 as a composite index reflecting adiposity and dyslipidemia, and previous studies have linked it to diabetes, mortality, and vascular outcomes [12–14, 17, 24–28]. However, most existing studies has been cross-sectional, retrospective, or focused primarily on baseline CMI. Our findings extend this evidence by demonstrating, in a large prospective cohort, that dynamic changes in CMI were associated with CVD risk independent of LDL-C levels. We also observed a non-linear association, with an inflection point at 0.42. This is broadly consistent with the current evidence that the association between CMI and cardiometabolic outcomes maynot be strictly linear. For instance, Zha et al. reported a non-linear association between CMI and diabetes risk, with an inflection point at 1.01 [19], Whereas Higashiyama et al. found a U-shaped association between CMI and ischemic stroke in community-dwelling adults aged 30–79 years, with the lowest risk observed at CMI values between 0.76 and 1.11 [14]. Differences in study population, outcome definitions, age structures, and baseline risk distributions may partly explain these varying thresholds. The present study provides a threshold of 0.42 for CVD risk stratification within middle-aged and older Chinese adults, which may serve as a practical reference for future research and potential clinical screening strategies.

Our findings should also be interpreted in the context of previous studies that have examined TG/HDL-C ratio or WHtR separately as longitudinal predictors of cardiovascular outcomes. These two markers reflect different but related aspects of cardiometabolic dysfunction, namely atherogenic dyslipidaemia and central adiposity. By integrating both components, CMI may provide a more comprehensive summary of metabolic risk than either indicator alone. In this regard, the present study extends the existing literature by evaluating not only baseline CMI but also multiple longitudinal CMI metrics, including change patterns, cumulative exposure, and absolute changes, in relation to CVD risk. Nevertheless, our study was not designed to formally compare the predictive performance of CMI with that of TG/HDL-C ratio or WHtR alone, and such head-to-head comparisons warrant further investigation.

Several mechanisms may help explain the observed associations. The TG/HDL-C ratio is a widely used surrogate marker of IR, and has been correlates with atherogenic lipoprotein phenotype, including smaller LDL particle size and greater remnant cholesterol burden, both of which have been linked toatherosclerotic cardiovascular disease even when LDL-C is within target range [29–31]. WHtR reflects central obesity and visceral fat depots in the abdominal cavity more sensitively than BMI or waist alone [32–34]. Visceral adiposity have been associated with adipokine dysregulation, elevated circulating free fatty acids, endothelial dysfunction, and inflammatory activation (e.g., IL-6 and hsCRP), all of which can accelerate atherogenesis and vascular remodeling [35, 36]. In our analysis, the association between CMI and CVD risk persisted after adjustment for hsCRP, suggesting that CMI may reflect residual cardiometabolic risk not fully captured by LDL-C and systemic inflammation.

Sex-stratified subgroup analyses indicated stronger associations in males than in females, which may reflect sex differences in visceral fat distribution, lipid metabolism and hormonal profiles [37]. We also found that elevated baseline CMI was associated withhigher CVD risk across hsCRP strata, with stronger associations observed in participants with higher hsCRP levels. These findings may suggest a potential synergistic effect between metabolic burden and systemic inflammation. Given growing evidence that chronic low-grade inflammation contributes to residual cardiovascular risk, these results support the view that combined assessment of metabolic indices (such as CMI) and inflammatory markers (such as hsCRP) may improve risk prediction in clinical practice [38].

Our findings highlight the potential importance of monitoring longitudinal changes in cardiometabolic status rather than relying on a single baseline assessments. Participants who progressed from low to high CMI reached a risk profile similar to those with persistently high CMI. Notably, in exploratory analyses, improvements in modifiable factors, such as weight normalization, increased physical activity, and healthier sleep patterns, were associated with a higher likelihood of CMI reduction whereas increasing hsCRP tended to accompany worsening CMI. While these observations do not establish causality, they support the potential utility of CMI as a responsive epidemiological marker that may complement conventional cardiovascular risk monitoring.

Emerging evidence has highlighted several approaches that may be relevant to residual cardiovascular risk by targeting metabolic domains of CMI. Current European guidelines continue to recommend stains as first-line therapy for patients with hypertriglyceridaemia [39], and earlier trials confirmed that fibrates can reduce TG and increase HDL-C levels by activating the selective peroxisome proliferator-activated receptor a (PPAR-a) [40, 41]. However, the PROMINENT trial recently demonstrated that the PPAR-a modulator pemafibrate did not reduce the risk of major adverse cardiovascular events in patients with diabetes, mild-to-moderate hypertriglyceridaemia and low HDL-C levels [42]. Moreover, a recent scientific statement underscores the crucial role of chronic, low-grade inflammation in CVD, noting that hsCRP *≥* 2 mg/L indicates active inflammation and is associated with increased CVD risk [43]. Consistent with this statement, recent clinical trials have highlighted the efficacy of anti-inflammatory approaches, including colchicine and canakinumab, in primary and secondary prevention of CVD [44]. These studies suggest that integrated management of lipid abnormalities, adiposity, and inflammation may contribute to CMI improvement.

This study has several strengths. To our knowledge, it is among the first large nationwide longitudinal prospective studies to systematically investigate baseline CMI together with multiple longitudinal CMI metrics, including change patterns, cumulative exposure and absolute changes, in relation to incident CVD. The CHARLS design provides standardized follow-up of health-related information, making extensive adjustment for cardiometabolic and inflammatory covariates possible. Multiple sensitivity analyses, including complete data analyses and inverse probability weighting, supported the robustness of the primary findings.This study also had some limitations. First, the ascertainment of CVD was based on self-reported physician-diagnosed information, which may introduce misclassification. Although this method is commonly used in large-scale cohort studies, self-reported diagnoses may be influenced by recall bias, variations in health awareness, and differential access to medical care. To enhance validity, CHARLS employs a serial verification protocol. In the next wave, respondents were required to confirm the presence of heart disease and stroke if they reported these conditions in the last wave. If the respondent disputed self-reported heart disease or stroke from previous waves, they were corrected retrospectively. Moreover, it is worth noting that highlight that other large-scale studies, like the English Longitudinal Study of Ageing and Health and Retirement Study, have confirmed a good concordance between self-reported stroke and medical records [45]. Nonetheless, some misclassification is unavoidable. To the extent that such misclassification was nondifferential, it would likely have biased the associations toward the null; however, differential misclassification cannot be ruled out, and this should be taken into account when interpreting the study findings. Second, selection bias may have occured because we excluded a large proportion of participants. However, IPW analyses indicated consistent results. Third, the study population consisted of middle-aged and older adults, limiting generalizability to younger population. Fourth, biomarkers used to calculate CMI were available at only two waves, limiting analyses of short-term variability and more complex trajectory patterns. Fifth, although we identified an apparent threshold of CMI, this value was derived and assessed within the same dataset without external validation. Therefore, it should be regarded as exploratory and may not be generalizable to other populations. Finally, the lack of additional inflammatory markers and detailed lipoprotein phenotyping limited an in-depth analysis of mechanistic analyses.

## Conclusions

In this large national prospective cohort study, baseline CMI and multiple longitudinal CMI metrics were associated with CVD risk, independent of LDL-C and hsCRP. The association between baseline CMI and CVD risk appeared to be nonlinear, and an inflection point was observed at CMI = 0.42 within this cohort. Importantly, participants whose CMI progressed from low to high had CVD risk comparable to those with persistently high CMI, whereas those with improvement from high to low CMI were not associated with excess risk compared with the stable low group. Among participants with low baseline CMI, cumulative CMI exposure and absolute change remainedstatistically significant, underscoring the potential relevanceof early identification and long-term monitoring of cardiometabolic burden. Collectively, these findings suggest that longitudinal CMI assessment may be useful for epidemiology risk stratification and highlight the potential value of CMI as a complementary tool for risk assessment in clinical and public health settings. However, as this is an observational study, it does not allow for causal inferences, and independent external validation is required before the proposed thresholds can be applied at the clinical or population level.

## Supplementary Information

Supplementary Material 1.

## Data Availability

Researchers can access the CHARLS data via the link [http://charls.pku.edu.cn/en/](http:/charls.pku.edu.cn/en) .

## Abbreviations

CVD: cardiovascular disease
CMI: cardiometabolic index
TG: triglycerides
HDL-C: high density lipoportein cholesterol
WHtR: waist-to-height ratio
CHARLS: China Health and Retirement Longitudinal Study
LDL-C: low-density lipoprotein cholesterol
hsCRP: high-sensitivity C-reactive protein
SBP: systolic blood pressure
DBP: diastolic blood pressure
HRs: hazard ratios
CIs: confidence intervals
IPW: inverse probability weighting
IR: insulin resistant
