# Supplementary material for: Longitudinal changes and cumulative exposure of cardiometabolic index predict cardiovascular disease risk in middle-aged and older adults: a national prospective cohort study
Source: BMC Cardiovasc Disord. 2026 Jun 27;26:794. doi: 10.1186/s12872-026-06101-3 (PMC13573330; doi:10.1186/s12872-026-06101-3)
Supplement: Supplementary file 1 [file 12872_2026_6101_MOESM1_ESM.docx]

**Supplementary Material**

Contents

[**Supplementary Methods**	2](#_Toc219900745)

[**Table S1. The missing number and rates of covariates.**	3](#_Toc219900746)

[**Table S2. Baseline characteristics of 9120 participants according to quartiles of baseline CMI**	4](#_Toc219900747)

[**Figure S1. Changes of CMI and its components from 2011 to 2015.**	5](#_Toc219900748)

[**Table S3. Associations of baseline CMI with clinical outcomes**	6](#_Toc219900749)

[**Figure S2. Confirmation of threshold by utilizing Maximally Selected Rank Statisitcs.**	7](#_Toc219900750)

[**Table S4. Associations of changes in CMI with heart diseases and stroke**	7](#_Toc219900751)

[**Table S5. Associations of cumulative CMI with heart diseases**	7](#_Toc219900752)

[**Table S6. Associations of cumulative CMI with stroke**	8](#_Toc219900753)

[**Table S7. Associations of ΔCMI with CVD**	9](#_Toc219900754)

[**Table S8. Associations of ΔCMI with heart diseases**	9](#_Toc219900755)

[**Table S9. Associations of ΔCMI with stroke**	10](#_Toc219900756)

[**Table S10. Associations of baseline CMI with CVD in complete dataset**	10](#_Toc219900757)

[**Table S11. Associations of changes in CMI with CVD in complete dataset**	10](#_Toc219900758)

[**Table S12. Associations of cumulative CMI with CVD in complete dataset**	11](#_Toc219900759)

[**Table S13. Associations of ΔCMI with CVD in complete dataset**	11](#_Toc219900760)

[**Table S14. Comparison of baseline characteristics of the excluded and included participants before and after inverse probability weighting**	12](#_Toc219900761)

[**Figure S3. Love plots show differences based on the original unweighted and inverse probability weighted participants.**	13](#_Toc219900762)

[**Table S15. Associations of changes in CMI with CVD with the use of inverse probability weighting (HR (95%CI))**	13](#_Toc219900763)

[**Table S16. Associations of cumulative CMI and ΔCMI with CVD with the use of inverse probability weighting (HR (95%CI))**	14](#_Toc219900764)

[**Table S17. Subgroup analysis of baseline CMI with clinical outcomes in participants with or without hypertension**	15](#_Toc219900765)

[**Table S18. Subgroup analysis of changes in CMI with clinical outcomes in participants with or without hypertension**	15](#_Toc219900766)

[**Table S19. Subgroup analysis of cumulative CMI with CVD in participants with or without hypertension**	16](#_Toc219900767)

[**Table S20. Subgroup analysis of ΔCMI with CVD in participants with or without hypertension**	16](#_Toc219900768)

[**Table S21. Subgroup analysis of baseline CMI with clinical outcomes in male and female**	17](#_Toc219900769)

[**Table S22. Subgroup analysis of changes in CMI with clinical outcomes in male and female**	18](#_Toc219900770)

[**Table S23. Subgroup analysis of cumulative CMI with CVD in male and female**	18](#_Toc219900771)

[**Table S24. Subgroup analysis of ΔCMI with CVD in male and female**	19](#_Toc219900772)

[**Table S25. Subgroup analysis of baseline CMI with clinical outcomes in different levels of CRP**	19](#_Toc219900773)

[**Table S26. Subgroup analysis of changes in CMI with clinical outcomes in different levels of CRP**	20](#_Toc219900774)

[**Table S27. Subgroup analysis of cumulative CMI with CVD in different levels of CRP**	21](#_Toc219900775)

[**Table S28. Subgroup analysis of ΔCMI with CVD in different levels of CRP**	21](#_Toc219900776)

[**Table S29. Changes of factors associated with CVD during follow-up in the participants with high baseline CMI level***	22](#_Toc219900777)

[**Table S30. Incremental value of adding changes in CMI to baseline model**	23](#_Toc219900778)

[**Figure S4. Decile-based calibration plots at 3-year.**	23](#_Toc219900779)

**Supplementary Methods**

**Covariates**

Education level was categorized into three levels: low (less than lower secondary), middle (upper secondary and vocational training), and high (tertiary). Smoking habits was classified into three categories: never, former and current smokers. The venous blood samples were collected from each participant by medically-trained staff from the China Center for Disease Control and Prevention (China CDC) in baseline and the third survey^1^. The samples of blood were kept under 4℃ until the samples were centrifuged at local hospitals or health center. Then, the samples were immediately stored frozen at -20℃ and were transferred to China CDC in Beijing within two weeks by a cold-chain shipping company. The levels of TC, TG and HDL-C were determined through enzymatic colormetric test. The high-sensitivity CRP levels were quantified using immunoturbidimetric assay, while serum creatinine was measured using rate-blanked and compensated Jaffe creatinine method. All measurements were conducted at Youanmen Center for Clinical Laboratory of Capital Medical University.

The physical examination collect at Wave 1 and Wave 3, and includes resting blood pressure (sitting) and anthropometric measures (weight, height and waist)^2^. Blood pressure was measured three times at 45-second intervals using an electronic sphygmomanometer (Omron HEM-7200), and the mean value of the last two readings was used in the analysis. Body weight and height in light clothing and without shoes were measured.

**Definitions of clinical outcomes**

Cardiovascular disease (CVD) was defined based on the self-reported physician-diagnosed heart diseases or stroke. In each wave of the CHARLS, participants were asked "Have you have been diagnosed with heart attack, coronary heart disease, angina, congestive heart failure, or other heart problems by a doctor?" and "Have you been diagnosed with stroke by a doctor?" Those who reported being diagnosed with heart diseases or stroke were considered to have CVD. In the next wave, the respondents were required to confirm the existence of heart disease and stroke if they reported those in the last wave. If the respondent disputed self-reported heart disease or stroke from previous waves, they were corrected retrospectively. Our CVD ascertainment was consistent with previous studies using the CHARLS^3^.

**Multiple imputation**

The missing data of covariates were imputed using the multiple imputation with chained equation^4^. The missing number and rates of covariates were showed in Table S1. We performed 5 imputations and generated 5 imputed datasets. Effect estimates were computed separately for each of the 5 datasets, and then combined according to Rubin’s rules. The multiple imputation was conducted using the R package “mice”.

**Table S1. The missing number and rates of covariates.**

| Variables | Smoking | Drinking | Lipid-lowering | Glucose | LDL-C | SBP | DBP | hsCRP |
| --- | --- | --- | --- | --- | --- | --- | --- | --- |
| No. (%) | 7 (0.1) | 12 (0.1) | 198 (2.2) | 9 (0.1) | 18 (0.2) | 88 (1.0) | 89 (1.0) | 1(0.01) |

There were no missing data of age, sex, education level, TG, HDL, waist and height.

**Competing-risks analyses**

Competing-risks regression was used to evaluate the association between baseline CMI and CVD. CVD was defined as the event of interest, while non-cardiovascular death was defined as a competing event. Sub-distribution hazard ratios (SHRs) and 95%CI were estimated using the Fine-Gray proportional sub-distribution hazards model. Cumulative incidence function using the Fine-Gray method. Cumulative incidence curves were plotted according to the quartiles of CMI.

**Table S2. Baseline characteristics of 9120 participants according to quartiles of baseline CMI**

|  | **Overall**  (n=9120) | **Baseline CMI** | | | | ***p*-value^1^** |
| --- | --- | --- | --- | --- | --- | --- |
|  |  | **Quartiles 1**  (n=2301) | **Quartiles 2**  (n=2239) | **Quartiles 3**  (n=2296) | **Quartiles4**  (n=2284) |  |
| Age, years | 59.3 (9.3) | 59.7 (9.6) | 59.4 (9.4) | 59.1 (9.2) | 58.8 (8.9) | 0.007 |
| Sex |  |  |  |  |  |  |
| male | 4257 (46.7) | 1275 (55.4) | 1072 (47.9) | 956 (41.6) | 954 (41.8) | <0.001 |
| female | 4863 (53.3) | 1026 (44.6) | 1167 (52.1) | 1340 (58.4) | 1330 (58.2) |  |
| BMI, *kg/m^2^* | 23.5 (3.9) | 21.4 (3.1) | 22.7 (3.5) | 24.2 (3.7) | 25.7 (3.9) | <0.001 |
| Waist, *cm* | 85.4 (10.1) | 79.2 (8.0) | 83.3 (8.8) | 87.2 (9.4) | 91.7 (9.5) | <0.001 |
| Height, *cm* | 157.9 (8.6) | 158.2 (8.5) | 157.7 (8.6) | 157.5 (8.7) | 158.1 (8.5) | 0.023 |
| SBP, *mmHg* | 129.7 (21.5) | 126.2 (21.2) | 127.6 (20.6) | 131.1 (21.8) | 133.8 (21.4) | <0.001 |
| DBP, *mmHg* | 75.4 (12.2) | 72.8 (12.0) | 74.3 (11.6) | 76.4 (12.4) | 78.0 (12.1) | <0.001 |
| TC, *mmol/L* | 5.0 (1.0) | 4.9 (0.9) | 4.9 (1.0) | 5.1 (1.0) | 5.3 (1.1) | <0.001 |
| TG, *mmol/L* | 1.5 (1.2) | 0.7 (0.2) | 1.0 (0.2) | 1.4 (0.3) | 2.8 (1.8) | <0.001 |
| HDL-C, *mmol/L* | 1.3 (0.4) | 1.7 (0.4) | 1.4 (0.3) | 1.2 (0.2) | 1.0 (0.2) | <0.001 |
| LDL-C, *mmol/L* | 3.0 (0.9) | 2.9 (0.8) | 3.1 (0.9) | 3.2 (0.9) | 2.9 (1.1) | <0.001 |
| Glucose, *mmol/L* | 6.1 (2.1) | 5.7 (1.4) | 5.8 (1.6) | 6.1 (2.0) | 6.9 (2.8) | <0.001 |
| WBC *in thousands* | 6.3 (2.2) | 6.0 (1.8) | 6.2 (1.9) | 6.4 (2.9) | 6.6 (1.9) | <0.001 |
| hsCRP, *mg/L* | 2.8 (7.6) | 2.7 (8.7) | 2.7 (6.9) | 2.7 (7.5) | 3.0 (7.2) | 0.395 |
| Smoking habit, n (%) | |  |  |  |  | <0.001 |
| never | 5521 (60.5) | 1247 (54.2) | 1331 (59.4) | 1492 (65.0) | 1451 (63.5) |  |
| former | 836 (9.2) | 213 (9.3) | 193 (8.6) | 201 (8.8) | 229 (10.0) |  |
| current | 2756 (30.2) | 839 (36.5) | 713 (31.8) | 603 (26.3) | 601 (26.3) |  |
| Drinking, n (%) |  |  |  |  |  | <0.001 |
| No | 5559 (61.0) | 1222 (53.1) | 1368 (61.1) | 1495 (65.1) | 1474 (64.5) |  |
| Yes | 3549 (38.9) | 1076 (46.8) | 869 (38.8) | 798 (34.8) | 806 (35.3) |  |
| Education level, n (%) | |  |  |  |  | 0.367 |
| Low | 8281 (90.8) | 2110 (91.7) | 2037 (91.0) | 2077 (90.5) | 2057 (90.1) |  |
| Middle | 743 (8.1) | 170 (7.4) | 182 (8.1) | 196 (8.5) | 195 (8.5) |  |
| High | 96 (1.1) | 21 (0.9) | 20 (0.9) | 23 (1.0) | 32 (1.4) |  |
| Medication use, n(%) | |  |  |  |  |  |
| Antidiabetic | 359 (3.9) | 53 (2.3) | 54 (2.4) | 86 (3.7) | 166 (7.3) | <0.001 |
| Antihypertension | 1804 (19.8) | 263 (11.4) | 333 (14.9) | 510 (22.2) | 698 (30.6) | <0.001 |
| Lipid-lowering | 465 (5.1) | 46 (2.0) | 80 (3.6) | 113 (4.9) | 226 (9.9) | <0.001 |
| Hypertension, n(%) |  |  |  |  |  |  |
| No | 5277 (57.9) | 1558 (67.7) | 1439 (64.3) | 1244 (54.2) | 1036 (45.4) | <0.001 |
| Yes | 3805 (41.7) | 733 (31.9) | 787 (35.1) | 1044 (45.5) | 1241 (54.3) |  |
| Diabetes, n(%) |  |  |  |  |  |  |
| No | 7541 (82.7) | 2081 (90.4) | 1962 (87.6) | 1911 (83.2) | 1587 (69.5) | <0.001 |
| Yes | 1579 (17.3) | 220 (9.6) | 277 (12.4) | 385 (16.8) | 697 (30.5) |  |

BMI, body mass index; BP, blood pressure; WBC, white blood cell; TC, total cholesterol; TG, triglyceride; HDL-C, high-density lipoprotein-cholesterol.

Data was expressed as mean (SD) for continuous variables and frequency (percentage) for categorical variables.

^1^ *p* values were obtained by ANOVA for continuous variables or the chi-square test for categorical variables.

**Figure S1. Changes of CMI and its components from 2011 to 2015.**

(A) the levels of CMI and its components in 2011 and 2015; (B) Mean CMI levels in 2011 and 2015 across four CMI change patterns; (C and D) Distribution of CMI in 2011 and 2015. TG, triglyceride; HDL-C, high-density lipoprotein-cholesterol; WHTR, waist to height ratio. ***, p<0.0001; **, p<0.01.

**Table S3. Associations of baseline CMI with clinical outcomes**

|  | **Quartile 1** | **Quartile 2** | **Quartile 3** | **Quartile 4** | ***P* for trend** |
| --- | --- | --- | --- | --- | --- |
| **CVD** | |  |  |  |  |
| Event proportion | 559/2301 (24.3) | 636/2239 (28.4) | 755/2296 (32.9) | 846/2284 (37.0) |  |
| Model 1 | Ref. | 1.22 (1.09, 1.37) | 1.42 (1.28, 1.59) | 1.64 (1.47, 1.82) | <0.001 |
| Model 2 | Ref. | 1.22 (1.09, 1.37) | 1.41 (1.26, 1.57) | 1.63 (1.46, 1.81) | <0.001 |
| Model 3 | Ref. | 1.15 (1.03, 1.29) | 1.26 (1.12, 1.41) | 1.33 (1.18, 1.48) | <0.001 |
| **Heart diseases** |  |  |  |  |  |
| Event proportion | 469/2301 (20.4) | 516/2239 (23.0) | 618/2296 (26.9) | 688/2284 (30.1) |  |
| Model 1 | Ref. | 1.17 (1.03, 1.33) | 1.37 (1.21, 1.54) | 1.55 (1.38, 1.74) | <0.001 |
| Model 2 | Ref. | 1.16 (1.02, 1.31) | 1.33 (1.18, 1.5) | 1.51 (1.34, 1.7) | <0.001 |
| Model 3 | Ref. | 1.09 (0.96, 1.24) | 1.19 (1.05, 1.34) | 1.24 (1.09, 1.4) | <0.001 |
| **Stroke** |  |  |  |  |  |
| Event proportion | 141/2301 (6.1) | 196/2239 (8.8) | 231/2296 (10.1) | 291/2284 (12.7) |  |
| Model 1 | Ref. | 1.46 (1.18, 1.82) | 1.68 (1.36, 2.07) | 2.17 (1.78, 2.66) | <0.001 |
| Model 2 | Ref. | 1.51 (1.22, 1.87) | 1.77 (1.44, 2.19) | 2.31 (1.88, 2.83) | <0.001 |
| Model 3 | Ref. | 1.41 (1.14, 1.76) | 1.52 (1.23, 1.89) | 1.78 (1.44, 2.19) | <0.001 |

Model 1: crude model;

Model 2: adjusted for age and sex;

Model 3: additionally adjusted for smoking habit, drinking, education level, LDL-C, glucose, SBP, DBP, hsCRP and medication use of lipid-lowering.

**Figure S2. Confirmation of threshold by utilizing Maximally Selected Rank Statisitcs.**

(Upper figure) Determination of cut point at 0.42. (Lower figure) Kaplan-Meier survival curves when cohort stratified as high (≥0.42) or low (<0.42).

**Table S4. Associations of changes in CMI with heart diseases and stroke**

|  | **Event proportion** | **Model 1** | | **Model 2** | |
| --- | --- | --- | --- | --- | --- |
|  |  | **HR (95%CI)** | ***P* value** | **HR (95%CI)** | ***P* value** |
| **Heart diseases** | | |  |  |  |
| Stable low | 271/1632 (16.6) | Ref. |  | Ref. |  |
| Low to high | 205/990 (20.7) | 1.22 (1.02, 1.47) | 0.03 | 1.23 (1.02, 1.47) | 0.029 |
| High to low | 124/629 (19.7) | 1.17 (0.95, 1.45) | 0.142 | 1.18 (0.95, 1.46) | 0.134 |
| Stable high | 706/2913 (24.2) | 1.29 (1.11, 1.49 | 0.001 | 1.3 (1.12, 1.52) | 0.001 |
| **Stroke** |  |  |  |  |  |
| Stable low | 85/1632 (5.2) | Ref. |  | Ref. |  |
| Low to high | 65/990 (6.6) | 1.29 (0.93, 1.79) | 0126 | 1.23 (0.93, 1.79) | 0.123 |
| High to low | 34/629 (5.4) | 1.01 (0.67, 1.50) | 0.978 | 1.01 (0.68, 1.51) | 0.953 |
| Stable high | 287/2913 (9.9) | 1.68 (1.31, 2.17) | <0.001 | 1.71 (1.31, 2.22) | <0.001 |

Model 1 adjusted for age, sex, smoking habit, drinking, education level, LDL-C, glucose, SBP, DBP, hsCRP and medication use of lipid-lowering.

Model 2 additionally adjusted for baseline CMI.

**Table S5. Associations of cumulative CMI with heart diseases**

|  | **Event proportion** | **Model 1** | | **Model 2** | |
| --- | --- | --- | --- | --- | --- |
|  |  | **HR (95%CI)** | ***P* value** | **HR (95%CI)** | ***P* value** |
| **Total population (n=6164)** | | |  |  |  |
| T1 | 464/2076 (22.4%) | Ref. |  | Ref. |  |
| T2 | 409/2040 (20%) | 0.95 (0.83, 1.09) | 0.477 | 0.96 (0.83, 1.1) | 0.564 |
| T3 | 433/2048 (21.1%) | 0.97 (0.85, 1.11) | 0.669 | 0.98 (0.85, 1.12) | 0.746 |
| P for trend |  | 0.664 |  | 0.757 |  |
| **Participants with low baseline CMI (<0.42) (n=2622)** | | | |  |  |
| T1 | 39/251 (15.5%) | Ref. |  | Ref. |  |
| T2 | 232/1330 (17.4%) | 1.05 (0.74, 1.47) | 0.797 | 1.02 (0.72, 1.44) | 0.930 |
| T3 | 205/1041 (19.7%) | 1.26 (0.89, 1.78) | 0.197 | 1.23 (0.86, 1.74) | 0.251 |
| P for trend |  | 0.056 |  | 0.062 |  |
| **Participants with high baseline CMI (≥0.42) (n=3542)** | | | |  |  |
| T1 | 425/1825 (23.3%) | Ref. |  | Ref. |  |
| T2 | 177/710 (24.9%) | 1.11 (0.93, 1.33) | 0.239 | 1.11 (0.93, 1.33) | 0.251 |
| T3 | 228/1007 (22.6%) | 0.93 (0.79, 1.10) | 0.384 | 0.93 (0.79, 1.10) | 0.383 |
| P for trend |  | 0.496 |  | 0.477 |  |

Model 1 adjusted for age, sex, smoking habit, drinking, education level, LDL-C, glucose, SBP, DBP, hsCRP and medication use of lipid-lowering.

Model 2 additionally adjusted for baseline CMI.

**Table S6. Associations of cumulative CMI with stroke**

|  | **Event proportion** | **Model 1** | | **Model 2** | |
| --- | --- | --- | --- | --- | --- |
|  |  | **HR (95%CI)** | ***P* value** | **HR (95%CI)** | ***P* value** |
| **Total population (n=6164)** | | |  |  |  |
| T1 | 173/2076 (8.3%) | Ref. |  | Ref. |  |
| T2 | 130/2040 (6.4%) | 0.84 (0.67, 1.06) | 0.138 | 0.86 (0.67, 1.09) | 0.198 |
| T3 | 168/2048 (8.2%) | 1.05 (0.85, 1.31) | 0.649 | 1.07 (0.86, 1.33) | 0.558 |
| P for trend |  | 0.660 |  | 0.529 |  |
| **Participants with low baseline CMI (<0.42) (n=2622)** | | | |  |  |
| T1 | 18/251 (7.2%) | Ref. |  | Ref. |  |
| T2 | 68/1330 (5.1%) | 0.68 (0.40, 1.15) | 0.154 | 0.78 (0.45, 1.33) | 0.360 |
| T3 | 64/1041 (6.1%) | 0.85 (0.50, 1.45) | 0.547 | 0.94 (0.55, 1.61) | 0.820 |
| P for trend |  | 0.864 |  | 0.727 |  |
| **Participants with high baseline CMI (≥0.42) (n=3542)** | | | |  |  |
| T1 | 155/1825 (8.5%) | Ref. |  | Ref. |  |
| T2 | 62/710 (8.7%) | 1.11 (0.82, 1.50) | 0.482 | 1.11 (0.82, 1.50) | 0.509 |
| T3 | 104/1007 (10.3%) | 1.27 (0.99, 1.64) | 0.065 | 1.27 (0.98, 1.64) | 0.074 |
| P for trend |  | 0.066 |  | 0.075 |  |

Model 1 adjusted for age, sex, smoking habit, drinking, education level, LDL-C, glucose, SBP, DBP, hsCRP and medication use of lipid-lowering.

Model 2 additionally adjusted for baseline CMI.

**Table S7. Associations of ΔCMI with CVD**

|  | **Event proportion** | **Model 1** | | **Model 2** | |
| --- | --- | --- | --- | --- | --- |
|  |  | **HR (95%CI)** | ***P* value** | **HR (95%CI)** | ***P* value** |
| **Total population (n=6164)** | | |  |  |  |
| ΔT1 | 554/2076 (26.7%) | 1.02 (0.90, 1.15) | 0.797 | 1.00 (0.88, 1.14) | 0.966 |
| ΔT2 | 468/2040 (22.9%) | 0.92 (0.81, 1.04) | 0.195 | 0.92 (0.81, 1.05) | 0.210 |
| ΔT3 | 520/2048 (25.4%) | Ref. |  | Ref. |  |
| P for trend |  | 0.200 |  | 0.217 |  |
| **Participants with low baseline CMI (<0.42) (n=2622)** | | | |  |  |
| ΔT1 | 48/251 (19.1%) | 0.82 (0.60, 1.12) | 0.212 | 0.82 (0.60, 1.13) | 0.225 |
| ΔT2 | 271/1330 (20.4%) | 0.82 (0.69, 0.98) | 0.026 | 0.82 (0.69, 0.98) | 0.025 |
| ΔT3 | 245/1041 (23.5%) | Ref. |  | Ref. |  |
| P for trend |  | 0.027 |  | 0.026 |  |
| **Participants with high baseline CMI (≥0.42) (n=3542)** | | | |  |  |
| ΔT1 | 506/1825 (27.7%) | 1.04 (0.89, 1.21) | 0.631 | 1.03 (0.89, 1.20) | 0.663 |
| ΔT2 | 197/710 (27.7%) | 1.07 (0.89, 1.29) | 0.454 | 1.07 (0.89, 1.29 | 0.450 |
| ΔT3 | 275/1007 (27.3%) | Ref. |  | Ref. |  |
| P for trend |  | 0.449 |  | 0.449 |  |

Model 1 adjusted for age, sex, smoking habit, drinking, education level, LDL-C, glucose, SBP, DBP, hsCRP at baseline and medication use of lipid-lowering.

Model 2 additionally adjusted for baseline CMI.

**Table S8. Associations of ΔCMI with heart diseases**

|  | **Event proportion** | **Model 1** | | **Model 2** | |
| --- | --- | --- | --- | --- | --- |
|  |  | **HR (95%CI)** | ***P* value** | **HR (95%CI)** | ***P* value** |
| **Total population (n=6164)** | | |  |  |  |
| ΔT1 | 464/2076 (22.4%) | 1.03 (0.90, 1.18) | 0.669 | 1.02 (0.89, 1.17) | 0.746 |
| ΔT2 | 409/2040 (20%) | 0.98 (0.86, 1.12) | 0.776 | 0.98 (0.86, 1.13) | 0.790 |
| ΔT3 | 433/2048 (21.1%) | Ref. |  | Ref. |  |
| P for trend |  | 0.781 |  | 0.799 |  |
| **Participants with low baseline CMI (<0.42) (n=2622)** | | | |  |  |
| ΔT1 | 39/251 (15.5%) | 0.80 (0.56, 1.13) | 0.197 | 0.81 (0.57, 1.16) | 0.251 |
| ΔT2 | 232/1330 (17.4%) | 0.83 (0.69, 1.01) | 0.060 | 0.83 (0.68, 1.00) | 0.052 |
| ΔT3 | 205/1041 (19.7%) | Ref. |  | Ref. |  |
| P for trend |  | 0.065 |  | 0.054 |  |
| **Participants with high baseline CMI (≥0.42) (n=3542)** | | | |  |  |
| ΔT1 | 425/1825 (23.3%) | 1.08 (0.91, 1.27) | 0.384 | 1.08 (0.91, 1.27) | 0.383 |
| ΔT2 | 177/710 (24.9%) | 1.20 (0.98, 1.46) | 0.074 | 1.20 (0.98, 1.46 | 0.075 |
| ΔT3 | 228/1007 (22.6%) | Ref. |  | Ref. |  |
| P for trend |  | 0.078 |  | 0.078 |  |

Model 1 adjusted for age, sex, smoking habit, drinking, education level, LDL-C, glucose, SBP, DBP, hsCRP and medication use of lipid-lowering.

Model 2 additionally adjusted for baseline CMI.

**Table S9. Associations of ΔCMI with stroke**

|  | **Event proportion** | **Model 1** | | **Model 2** | |
| --- | --- | --- | --- | --- | --- |
|  |  | **HR (95%CI)** | ***P* value** | **HR (95%CI)** | ***P* value** |
| **Total population (n=6164)** | | |  |  |  |
| ΔT1 | 173/2076 (8.3%) | 0.95 (0.77, 1.18) | 0.649 | 0.94 (0.75, 1.17) | 0.558 |
| ΔT2 | 130/2040 (6.4%) | 0.80 (0.63, 1.01) | 0.057 | 0.80 (0.63, 1.01) | 0.060 |
| ΔT3 | 168/2048 (8.2%) | Ref. |  | Ref. |  |
| P for trend |  | 0.059 |  | 0.062 |  |
| **Participants with low baseline CMI (<0.42) (n=2622)** | | | |  |  |
| ΔT1 | 18/251 (7.2%) | 1.18 (0.69, 2.01) | 0.547 | 1.06 (0.62, 1.83) | 0.820 |
| ΔT2 | 68/1330 (5.1%) | 0.80 (0.57, 1.14) | 0.218 | 0.83 (0.58, 1.17) | 0.286 |
| ΔT3 | 64/1041 (6.1%) | Ref. |  | Ref. |  |
| P for trend |  | 0.204 |  | 0.282 |  |
| **Participants with high baseline CMI (≥0.42) (n=3542)** | | | |  |  |
| ΔT1 | 155/1825 (8.5%) | 0.79 (0.61, 1.02) | 0.065 | 0.79 (0.61, 1.02) | 0.074 |
| ΔT2 | 62/710 (8.7%) | 0.88 (0.64, 1.20) | 0.411 | 0.87 (0.64, 1.20) | 0.409 |
| ΔT3 | 104/1007 (10.3%) | Ref. |  | Ref. |  |
| P for trend |  | 0.285 |  | 0.287 |  |

Model 1 adjusted for age, sex, smoking habit, drinking, education level, LDL-C, glucose, SBP, DBP, hsCRP and medication use of lipid-lowering.

Model 2 additionally adjusted for baseline CMI.

**Table S10. Associations of baseline CMI with CVD in complete dataset**

|  | **Quartile 1** | **Quartile 2** | **Quartile 3** | **Quartile 4** | ***P* for trend** |
| --- | --- | --- | --- | --- | --- |
| Model 1 | Ref. | 1.22 (1.09, 1.37) | 1.42 (1.28, 1.59) | 1.64 (1.47, 1.82) | <0.001 |
| Model 2 | Ref. | 1.22 (1.09, 1.37) | 1.41 (1.26, 1.57) | 1.63 (1.46, 1.81) | <0.001 |
| Model 3 | Ref. | 1.15 (1.02, 1.29) | 1.25 (1.12, 1.41) | 1.32 (1.18, 1.48) | <0.001 |

**Model 1:** crude model;

**Model 2:** adjusted for age and sex;

**Model 3:** additionally adjusted for smoking habit, drinking, education level, LDL-C, glucose, SBP, DBP, hsCRP and medication use of lipid-lowering.

**Table S11. Associations of changes in CMI with CVD in complete dataset**

|  | **Model 1** | | **Model 2** | |
| --- | --- | --- | --- | --- |
|  | **HR (95%CI)** | ***P* value** | **HR (95%CI)** | ***P* value** |
| Stable low | Ref. |  | Ref. |  |
| Low to high | 1.24 (1.05, 1.47) | 0.012 | 1.24 (1.05, 1.47) | 0.013 |
| High to low | 1.13 (0.93, 1.39) | 0.217 | 1.13 (0.92, 1.38) | 0.133 |
| Stable high | 1.31 (1.14, 1.50) | <0.001 | 1.28 (1.11, 1.49) | <0.001 |

Model 1 adjusted for age, sex, smoking habit, drinking, education level, LDL-C, glucose, SBP, DBP, hsCRP and medication use of lipid-lowering.

Model 2 additionally adjusted for baseline CMI.

**Table S12. Associations of cumulative CMI with CVD in complete dataset**

|  | **Model 1** | | **Model 2** | |
| --- | --- | --- | --- | --- |
|  | **HR (95%CI)** | ***P* value** | **HR (95%CI)** | ***P* value** |
| **Total population (n=6164)** | | | | |
| T1 | Ref. |  | Ref. |  |
| T2 | 0.89 (0.78, 1.01) | 0.069 | 0.92 (0.80, 1.05) | 0.217 |
| T3 | 0.97 (0.86, 1.10) | 0.635 | 1.00 (0.88, 1.13) | 0.967 |
| P for trend | 0.620 |  | 0.977 |  |
| **Participants with low baseline CMI (<0.42)** | | | | |
| T1 | Ref. |  | Ref. |  |
| T2 | 0.97 (0.71, 1.33) | 0.833 | 0.97 (0.70, 1.33) | 0.832 |
| T3 | 1.18 (0.86, 1.62) | 0.315 | 1.18 (0.85, 1.63) | 0.323 |
| P for trend | 0.061 |  | 0.060 |  |
| **Participants with high baseline CMI (≥0.42)** | | | | |
| T1 | Ref. |  | Ref. |  |
| T2 | 1.01 (0.85, 1.19) | 0.942 | 1.02 (0.86, 1.22) | 0.806 |
| T3 | 0.95 (0.82, 1.11) | 0.513 | 0.96 (0.82, 1.12) | 0.628 |
| P for trend | 0.540 |  | 0.659 |  |

Model 1 adjusted for age, sex, smoking habit, drinking, education level, LDL-C, glucose, SBP, DBP, hsCRP and medication use of lipid-lowering.

Model 2 additionally adjusted for baseline CMI.

**Table S13. Associations of ΔCMI with CVD in complete dataset**

|  | **Model 1** | | **Model 2** | | |
| --- | --- | --- | --- | --- | --- |
|  | **HR (95%CI)** | ***P* value** | **HR (95%CI)** | ***P* value** | |
| **Total population (n=6164)** | | | | |  |
| ΔT1 | 1.03 (0.91, 1.17) | 0.635 | 1.00 (0.88, 1.14) | 0.967 | |
| ΔT2 | 0.92 (0.81, 1.04) | 0.181 | 0.92 (0.81, 1.05) | 0.213 | |
| ΔT3 | Ref. |  | Ref. |  | |
| P for trend | 0.188 |  | 0.223 |  | |
| **Participants with low baseline CMI (<0.42)** | | | | |  |
| ΔT1 | 0.85 (0.62, 1.17) | 0.315 | 0.85 (0.61, 1.17) | 0.323 | |
| ΔT2 | 0.82 (0.69, 0.98) | 0.03 | 0.82 (0.69, 0.98) | 0.03 | |
| ΔT3 | Ref. |  | Ref. |  | |
| P for trend | 0.031 |  | 0.031 |  | |
| **Participants with high baseline CMI (≥0.42)** | | | | |  |
| ΔT1 | 1.05 (0.90, 1.23) | 0.513 | 1.04 (0.89, 1.21) | 0.628 | |
| ΔT2 | 1.06 (0.88, 1.28) | 0.552 | 1.06 (0.88, 1.28) | 0.531 | |
| ΔT3 | Ref. |  | Ref. |  | |
| P for trend | 0.521 |  | 0.517 |  | |

Model 1 adjusted for age, sex, smoking habit, drinking, education level, LDL-C, glucose, SBP, DBP, hsCRP and medication use of lipid-lowering.

Model 2 additionally adjusted for baseline CMI.

**Table S14. Comparison of baseline characteristics of the excluded and included participants before and after** **inverse probability weighting**

|  | **Participants** | | | **Before IPW** | | **After IPW** | |
| --- | --- | --- | --- | --- | --- | --- | --- |
|  | **Overall**  (n=17708) | **Excluded**  (n=11544) | **Included**  (n=6164) | ***P* value** | **SMD** | ***P* value** | **SMD** |
| Age, years | 58.50 (10.17) | 58.33 (10.90) | 58.80 (8.67) | 0.003 | 0.048 | 0.657 | 0.007 |
| Sex |  |  |  | 0.001 | 0.060 | 0.637 | 0.008 |
| male | 8478 (47.9) | 5641 (48.9) | 2837 (46.0) |  |  |  |  |
| female | 9228 (52.1) | 5901 (51.1) | 3327 (54.0) |  |  |  |  |
| Smoking habits |  |  |  | <0.001 | 0.215 | 0.733 | 0.013 |
| never | 10619 (60.0) | 6842 (59.3) | 3777 (61.3) |  |  |  |  |
| former | 2069 (11.7) | 1534 (13.3) | 535 (8.7) |  |  |  |  |
| current | 4871 (27.5) | 3024 (26.2) | 1847 (30.0) |  |  |  |  |
| Drinking, n (%) |  |  |  | <0.001 | 0.145 | 0.849 | 0.003 |
| No | 10718 (60.5) | 6946 (60.2) | 3772 (61.2) |  |  |  |  |
| Yes | 6823 (38.5) | 4439 (38.5) | 2384 (38.7) |  |  |  |  |
| Educational level |  |  |  | <0.001 | 0.200 | 0.001 | 0.065 |
| Low | 15545 (87.8) | 9904 (85.8) | 5641 (91.5) |  |  |  |  |
| Middle | 1793 (10.1) | 1323 (11.5) | 470 (7.6) |  |  |  |  |
| High | 354 (2.0) | 301 (2.6) | 53 (0.9) |  |  |  |  |
| Antidiabetic |  |  |  | <0.001 | 0.084 | 0.913 | 0.002 |
| No | 16692 (94.3) | 10827 (93.8) | 5865 (95.1) |  |  |  |  |
| Yes | 712 (4.0) | 478 (4.1) | 234 (3.8) |  |  |  |  |
| Antihypertension |  |  |  | <0.001 | 0.106 | 0.933 | 0.001 |
| No | 14046 (79.3) | 9140 (79.2) | 4906 (79.6) |  |  |  |  |
| Yes | 3414 (19.3) | 2196 (19.0) | 1218 (19.8) |  |  |  |  |
| Lipid-lowering |  |  |  | <0.001 | 0.073 | 0.982 | <0.001 |
| No | 16336 (92.3) | 10651 (92.3) | 5685 (92.2) |  |  |  |  |
| Yes | 865 (4.9) | 524 (4.5) | 341 (5.5) |  |  |  |  |
| hsCRP, *mg/L* | 2.80 (7.63) | 3.14 (8.44) | 2.50 (6.82) | <0.001 | 0.084 | 0.447 | 0.015 |

IPW: inverse probability weighting; SMD: standardized mean differences.

**Figure S3. Love plots show differences based on the original unweighted and inverse probability weighted participants.**

**Table S15. Associations of changes in CMI with CVD with the use of inverse probability weighting (HR (95%CI))**

|  | **Model 1** | | **Model 2** | |
| --- | --- | --- | --- | --- |
|  | **HR (95%CI)** | ***P* value** | **HR (95%CI)** | ***P* value** |
| **Changes in CMI** |  |  |  |  |
| Stable low | Ref. |  | Ref. |  |
| Low to high | 1.25 (1.06, 1.48) | 0.009 | 1.23 (1.04, 1.45) | 0.014 |
| High to low | 1.10 (0.90, 1.34) | 0.357 | 1.09 (0.90, 1.34) | 0.376 |
| Stable high | 1.30 (1.14, 1.49) | 0.0001 | 1.28 (1.12, 1.47) | 0.0004 |

Model 1 adjusted for age, sex, smoking habit, drinking, education level, LDL-C, glucose, SBP, DBP, hsCRP and medication use of lipid-lowering.

Model 2 additionally adjusted for baseline CMI.

**Table S16. Associations of cumulative CMI and ΔCMI with CVD with the use of inverse probability weighting (HR (95%CI))**

|  | **Model 1** | | **Model 2** | |
| --- | --- | --- | --- | --- |
|  | **HR (95%CI)** | ***P* value** | **HR (95%CI)** | ***P* value** |
| **Cumulative CMI** |  |  |  |  |
| **Overall** |  |  |  |  |
| T1 | Ref. |  | Ref. |  |
| T2 | 0.96 (0.86, 1.08) | 0.511 | 0.98 (0.88, 1.10) | 0.735 |
| T3 | 1.02 (0.92, 1.14) | 0.655 | 1.04 (0.93, 1.16) | 0.480 |
| **Participants with low baseline CMI** | |  |  |  |
| T1 | Ref. |  | Ref. |  |
| T2 | 1.14 (0.86, 1.50) | 0.361 | 1.17 (0.88, 1.55) | 0.276 |
| T3 | 1.33 (1.01, 1.76) | 0.044 | 1.37 (1.03, 1.82) | 0.031 |
| **Participants with high baseline CMI** | |  |  |  |
| T1 | Ref. |  | Ref. |  |
| T2 | 1.15 (0.99, 1.32) | 0.064 | 1.15 (0.99, 1.33) | 0.065 |
| T3 | 1.03 (0.91, 1.18) | 0.610 | 1.04 (0.91, 1.18) | 0603 |
| **ΔCMI** |  |  |  |  |
| **Overall** |  |  |  |  |
| ΔT3 | Ref. |  | Ref. |  |
| ΔT2 | 0.94 (0.84, 1.05) | 0.279 | 0.94 (0.86, 1.07) | 0.297 |
| ΔT1 | 0.98 (0.88, 1.09) | 0.655 | 0.96 (0.84, 1.05) | 0.480 |
| **Participants with low baseline CMI** | |  |  |  |
| ΔT3 | Ref. |  | Ref. |  |
| ΔT2 | 0.85 (0.73, 1.00) | 0.044 | 0.85 (0.73, 1.00) | 0.046 |
| ΔT1 | 0.75 (0.57, 0.99) | 0.044 | 0.73 (0.55, 0.97) | 0.031 |
| **Participants with high baseline CMI** | |  |  |  |
| ΔT3 | Ref. |  | Ref. |  |
| ΔT2 | 1.11 (0.94, 1.30) | 0.209 | 1.11 (0.94, 1.30) | 0.209 |
| ΔT1 | 0.97 (0.85, 1.10) | 0.610 | 0.97 (0.85, 1.10) | 0.603 |

Model 1 adjusted for age, sex, smoking habit, drinking, education level, LDL-C, glucose, SBP, DBP, hsCRP and medication use of lipid-lowering.

Model 2 additionally adjusted for baseline CMI.

**Table S17. Subgroup analysis of baseline CMI with clinical outcomes in participants with or without hypertension**

|  | **Quartile 1** | **Quartile 2** | **Quartile 3** | **Quartile 4** | ***P* for trend** |
| --- | --- | --- | --- | --- | --- |
| **CVD** | |  |  |  |  |
| **Normotension** |  |  |  |  |  |
| Event proportion | 305/1558 (19.6) | 332/1439 (23.1) | 319/1244 (25.6) | 283/1036 (27.3) |  |
| HR (95%CI) | Ref. | 1.16 (1.00, 1.36) | 1.29 (1.10, 1.51) | 1.36 (1.15, 1.61) | <0.001 |
| **Hypertension** |  |  |  |  |  |
| Event proportion | 249/733 (34.0) | 303/787 (38.5) | 434/1044 (41.6) | 563/1241 (45.4) |  |
| HR (95%CI) | Ref. | 1.12 (0.95, 1.33) | 1.15 (0.98, 1.34) | 1.18 (1.01, 1.37) | 0.045 |
| **Heart diseases** | |  |  |  |  |
| **Normotension** |  |  |  |  |  |
| Event proportion | 309/1566 (19.7) | 333/1446 (23.0) | 321/1250 (25.7) | 283/1042 (27.2) |  |
| HR (95%CI) | Ref. | 1.13 (0.96, 1.35) | 1.31 (1.1, 1.56) | 1.29 (1.07, 1.55) | 0.001 |
| **Hypertension** |  |  |  |  |  |
| Event proportion | 250/735 (34.0) | 303/793 (38.2) | 434/1046 (41.5) | 563/1242 (45.3) |  |
| HR (95%CI) | Ref. | 1.03 (0.86, 1.24) | 1.01 (0.85, 1.2) | 1.06 (0.89, 1.26) | 0.522 |
| **Stroke** | |  |  |  |  |
| **Normotension** |  |  |  |  |  |
| Event proportion | 64/1566 (4.1) | 85/1446 (5.9) | 76/1250 (6.1) | 68/1042 (6.5) |  |
| HR (95%CI) | Ref. | 1.46 (1.05, 2.02) | 1.50 (1.07, 2.11) | 1.63 (1.15, 2.32) | 0.007 |
| **Hypertension** |  |  |  |  |  |
| Event proportion | 77/735 (10.5) | 111/793 (14.0) | 155/1046 (14.8) | 223/1242 (18.0) |  |
| HR (95%CI) | Ref. | 1.37 (1.02, 1.84) | 1.42 (1.07, 1.88) | 1.65 (1.26, 2.17) | <0.001 |

Model adjusted for age, sex, smoking habit, drinking, education level, LDL-C, glucose, SBP, DBP, hsCRP and medication use of lipid-lowering.

**Table S18. Subgroup analysis of changes in CMI with clinical outcomes in participants with or without hypertension**

|  | **Stable low** | **Low to high** | **High to low** | **Stable high** |
| --- | --- | --- | --- | --- |
| **CVD** | |  |  |  |
| **Normotension** |  |  |  |  |
| Event proportion | 191/1146 (16.7) | 128/651 (19.7) | 63/383 (16.4) | 314/1433 (21.9) |
| HR (95%CI) | Ref. | 1.19 (0.95, 1.49) | 1.06 (0.79, 1.41) | 1.33 (1.10, 1.60) |
| **Hypertension** |  |  |  |  |
| Event proportion | 132/486 (36.5) | 113/339 (33.3) | 79/246 (32.1) | 522/1480 (35.3) |
| HR (95%CI) | Ref. | 1.26 (0.98, 1.62) | 1.11 (0.84, 1.47) | 1.16 (0.95, 1.41) |
| **Heart diseases** | |  |  |  |
| **Normotension** |  |  |  |  |
| Event proportion | 159/1146 (13.9) | 102/651 (15.7) | 55/383 (14.4) | 270/1433 (18.8) |
| HR (95%CI) | Ref. | 1.10 (0.86, 1.42) | 1.11 (0.81, 1.50) | 1.34 (1.09, 1.64) |
| **Hypertension** |  |  |  |  |
| Event proportion | 112/486 (23.0) | 103/339 (30.4) | 69/246 (28.0) | 436/1480 (29.5) |
| HR (95%CI) | Ref. | 1.33 (1.01, 1.74) | 1.14 (0.84, 1.54) | 1.12 (0.91, 1.39) |
| **Stroke** | |  |  |  |
| **Normotension** |  |  |  |  |
| Event proportion | 42/1146 (3.7) | 36/651 (5.5) | 15/383 (3.9) | 75/1433 (5.2) |
| HR (95%CI) | Ref. | 1.61 (1.02, 2.53) | 1.17 (0.64, 2.11) | 1.50 (1.01, 2.21) |
| **Hypertension** |  |  |  |  |
| Event proportion | 43/486 (8.8) | 29/339 (8.6) | 19/246 (7.7) | 212/1480 (14.3) |
| HR (95%CI) | Ref. | 1.02 (0.64, 1.65) | 0.87 (0.51, 1.50) | 1.57 (1.12, 2.21) |

Model adjusted for age, sex, smoking habit, drinking, education level, LDL-C, glucose, SBP, DBP, hsCRP and medication use of lipid-lowering.

**Table S19. Subgroup analysis of cumulative CMI with CVD in participants with or without hypertension**

|  | **T1** | **T2** | **T3** | **P for trend** |
| --- | --- | --- | --- | --- |
| **Participants with low baseline CMI** | |  |  |  |
| **Normotension** |  |  |  |  |
| Event proportion | 26/189 (13.8%) | 161/911 (17.7%) | 132/697 (18.9%) |  |
| HR (95%CI) | Ref. | 1.18 (0.78, 1.79) | 1.35 (0.88, 2.07) | 0.115 |
| **Hypertension** |  |  |  |  |
| Event proportion | 22/62 (35.5%) | 110/419 (26.3%) | 113/344 (32.8%) |  |
| HR (95%CI) | Ref. | 0.70 (0.44, 1.12) | 0.92 (0.58, 1.48) | 0.346 |
| **Participants with high baseline CMI** | |  |  |  |
| **Normotension** |  |  |  |  |
| Event proportion | 193/939 (20.6%) | 82/358 (22.9%) | 102/519 (19.7%) |  |
| HR (95%CI) | Ref. | 1.05 (0.81, 1.37) | 0.88 (0.68, 1.12) | 0.337 |
| **Hypertension** |  |  |  |  |
| Event proportion | 313/886 (35.3%) | 115/352 (32.7%) | 173/488 (35.5%) |  |
| HR (95%CI) | Ref. | 1.02 (0.82, 1.27) | 1.01 (0.83, 1.22) | 0.920 |

Model adjusted for age, sex, smoking habit, drinking, education level, LDL-C, glucose, SBP, DBP, hsCRP and medication use of lipid-lowering.

**Table S20. Subgroup analysis of ΔCMI with CVD in participants with or without hypertension**

|  | **ΔT1** | **ΔT2** | **ΔT3** | **P for trend** |
| --- | --- | --- | --- | --- |
| **Participants with low baseline CMI** | |  |  |  |
| **Normotension** |  |  |  |  |
| Event proportion | 26/189 (13.8%) | 161/911 (17.7%) | 132/697 (18.9%) |  |
| HR (95%CI) | 0.74 (0.48, 1.13) | 0.87 (0.69, 1.1) | Ref. | 0.280 |
| **Hypertension** |  |  |  |  |
| Event proportion | 22/62 (35.5%) | 110/419 (26.3%) | 113/344 (32.8%) |  |
| HR (95%CI) | 1.08 (0.68, 1.73) | 0.76 (0.58, 1.00) | Ref. | 0.045 |
| **Participants with high baseline CMI** | |  |  |  |
| **Normotension** |  |  |  |  |
| Event proportion | 193/939 (20.6%) | 82/358 (22.9%) | 102/519 (19.7%) |  |
| HR (95%CI) | 1.14 (0.89, 1.46) | 1.2 (0.9, 1.61) | Ref. | 0.205 |
| **Hypertension** |  |  |  |  |
| Event proportion | 313/886 (35.3%) | 115/352 (32.7%) | 173/488 (35.5%) |  |
| HR (95%CI) | 1.02 (0.8, 1.29 | 1.02 (0.8, 1.29 | Ref. | 0.921 |

Model adjusted for age, sex, smoking habit, drinking, education level, LDL-C, glucose, SBP, DBP, hsCRP and medication use of lipid-lowering.

**Table S21. Subgroup analysis of baseline CMI with clinical outcomes in male and female**

|  | **Quartile 1** | **Quartile 2** | **Quartile 3** | **Quartile 4** | ***P* for trend** |
| --- | --- | --- | --- | --- | --- |
| **CVD** | |  |  |  |  |
| **Male** |  |  |  |  |  |
| Event proportion | 291/1275 (22.8) | 266/1072 (24.8) | 284/956 (29.7) | 324/954 (34.0) |  |
| HR (95%CI) | Ref. | 1.05 (0.89, 1.24) | 1.28 (1.08, 1.51) | 1.41 (1.19, 1.66) | <0.001 |
| **Female** |  |  |  |  |  |
| Event proportion | 268/1026 (26.1) | 370/1167 (31.7) | 471/1340 (35.1) | 522/1330 (39.2) |  |
| HR (95%CI) | Ref. | 1.23 (1.05, 1.44) | 1.26 (1.08, 1.47) | 1.30 (1.12, 1.52) | 0.002 |
| **Heart diseases** | |  |  |  |  |
| **Male** |  |  |  |  |  |
| Event proportion | 233/1275 (18.3) | 206/1072 (19.2) | 224/956 (23.4) | 239/954 (25.1) |  |
| HR (95%CI) | Ref. | 1.01 (0.83, 1.21) | 1.24 (1.03, 1.5) | 1.27 (1.05, 1.54) | 0.003 |
| **Female** |  |  |  |  |  |
| Event proportion | 236/1026 (23.0) | 310/1167 (26.6) | 394/1340 (29.4) | 449/1330 (33.8) |  |
| HR (95%CI) | Ref. | 1.15 (0.97, 1.36) | 1.18 (1.00, 1.38) | 1.25 (1.06, 1.47) | 0.012 |
| **Stroke** | |  |  |  |  |
| **Male** |  |  |  |  |  |
| Event proportion | 89/1275 (7.0) | 99/1072 (9.2) | 101/956 (10.6) | 135/954 (14.2) |  |
| HR (95%CI) | Ref. | 1.25 (0.93, 1.66) | 1.40 (1.04, 1.87) | 1.77 (1.33, 2.34) | <0.001 |
| **Female** |  |  |  |  |  |
| Event proportion | 52/1026 (5.1) | 97/1167 (8.3) | 130/1340 (9.7) | 156/1330 (11.7) |  |
| HR (95%CI) | Ref. | 1.67 (1.19, 2.34) | 1.75 (1.26, 2.43) | 1.92 (1.39, 2.65) | <0.001 |

Model adjusted for age, smoking habit, drinking, education level, LDL-C, glucose, SBP, DBP, hsCRP and medication use of lipid-lowering.

**Table S22. Subgroup analysis of changes in CMI with clinical outcomes in male and female**

|  | **Stable low** | **Low to high** | **High to low** | **Stable high** |
| --- | --- | --- | --- | --- |
| **CVD** | |  |  |  |
| **Male** |  |  |  |  |
| Event proportion | 162/908 (17.8) | 93/433 (21.5) | 63/320 (19.7) | 317/1176 (27.0) |
| HR (95%CI) | Ref. | 1.31 (1.01, 1.69) | 1.14 (0.85, 1.52) | 1.50 (1.23, 1.83) |
| **Female** |  |  |  |  |
| Event proportion | 161/724 (22.2) | 148/557 (26.6) | 79/309 (25.6) | 519/1737 (29.9) |
| HR (95%CI) | Ref. | 1.14 (0.91, 1.43) | 1.09 (0.83, 1.43) | 1.16 (0.96, 1.39) |
| **Heart diseases** | |  |  |  |
| **Male** |  |  |  |  |
| Event proportion | 131/908 (14.4) | 77/433 (17.8) | 51/320 (15.9) | 258/1176 (21.9) |
| HR (95%CI) | Ref. | 1.34 (1.01, 1.79) | 1.13 (0.81, 1.56) | 1.51 (1.22, 1.88) |
| **Female** |  |  |  |  |
| Event proportion | 140/724 (19.3) | 128/557 (23.0) | 73/309 (23.6) | 448/1737 (25.8) |
| HR (95%CI) | Ref. | 1.13 (0.89, 1.43) | 1.18 (0.89, 1.57) | 1.14 (0.93, 1.38) |
| **Stroke** | |  |  |  |
| **Male** |  |  |  |  |
| Event proportion | 49/908 (5.4) | 30/433 (6.9) | 21/320 (6.6) | 119/1176 (10.1) |
| HR (95%CI) | Ref. | 1.31 (0.83, 2.07) | 1.24 (0.74, 2.07) | 1.69 (1.20, 2.38) |
| **Female** |  |  |  |  |
| Event proportion | 36/724 (5.0) | 35/557 (6.3) | 13/309 (4.2) | 168/1737 (9.7) |
| HR (95%CI) | Ref. | 1.22 (0.76, 1.96) | 0.78 (0.41, 1.49) | 1.66 (1.14, 2.42) |

Model adjusted for age, smoking habit, drinking, education level, LDL-C, glucose, SBP, DBP, hsCRP and medication use of lipid-lowering.

**Table S23. Subgroup analysis of cumulative CMI with CVD in male and female**

|  | **T1** | **T2** | **T3** | **P for trend** |
| --- | --- | --- | --- | --- |
| **Participants with low baseline CMI** | |  |  |  |
| **Male** |  |  |  |  |
| Event proportion | 24/153 (15.7%) | 127/711 (17.9%) | 104/477 (21.8%) |  |
| HR (95%CI) | Ref. | 1.07 (0.69, 1.66) | 1.48 (0.95, 2.32) | 0.015 |
| **Female** |  |  |  |  |
| Event proportion | 24/98 (24.5%) | 144/619 (23.3%) | 141/564 (25%) |  |
| HR (95%CI) | Ref. | 0.86 (0.56, 1.34) | 0.95 (0.62, 1.48) | 0.723 |
| **Participants with high baseline CMI** | |  |  |  |
| **Male** |  |  |  |  |
| Event proportion | 198/812 (24.4%) | 83/295 (28.1%) | 99/389 (25.4%) |  |
| HR (95%CI) | Ref. | 1.28 (0.99, 1.67) | 1.09 (0.85, 1.4) | 0.339 |
| **Female** |  |  |  |  |
| Event proportion | 176/618 (28.5%) | 176/618 (28.5%) | 176/618 (28.5%) |  |
| HR (95%CI) | Ref. | 0.90 (0.72, 1.11) | 0.90 (0.74, 1.09) | 0.244 |

Model adjusted for age, smoking habit, drinking, education level, LDL-C, glucose, SBP, DBP, hsCRP and medication use of lipid-lowering.

**Table S24. Subgroup analysis of ΔCMI with CVD in male and female**

|  | **ΔT1** | **ΔT2** | **ΔT3** | **P for trend** |
| --- | --- | --- | --- | --- |
| **Participants with low baseline CMI** | |  |  |  |
| **Male** |  |  |  |  |
| Event proportion | 24/153 (15.7%) | 127/711 (17.9%) | 104/477 (21.8%) |  |
| HR (95%CI) | 0.67 (0.43, 1.05) | 0.72 (0.55, 0.94) | Ref. | 0.018 |
| **Female** |  |  |  |  |
| Event proportion | 24/98 (24.5%) | 144/619 (23.3%) | 141/564 (25%) |  |
| HR (95%CI) | 1.05 (0.68, 1.63) | 0.91 (0.72, 1.15) | Ref. | 0.412 |
| **Participants with high baseline CMI** | |  |  |  |
| **Male** |  |  |  |  |
| Event proportion | 198/812 (24.4%) | 83/295 (28.1%) | 99/389 (25.4%) |  |
| HR (95%CI) | 0.92 (0.72, 1.17) | 1.18 (0.87, 1.58) | Ref. | 0.361 |
| **Female** |  |  |  |  |
| Event proportion | 308/1013 (30.4%) | 114/415 (27.5%) | 176/618 (28.5%) |  |
| HR (95%CI) | 1.11 (0.92, 1.35) | 1.00 (0.79, 1.27) | Ref. | 0.873 |

Model adjusted for age, smoking habit, drinking, education level, LDL-C, glucose, SBP, DBP, hsCRP and medication use of lipid-lowering.

**Table S25. Subgroup analysis of baseline CMI with clinical outcomes in different levels of CRP**

|  | **Quartile 1** | **Quartile 2** | **Quartile 3** | **Quartile 4** | ***P* for trend** |
| --- | --- | --- | --- | --- | --- |
| **CVD** | |  |  |  |  |
| **CRP ≤ 1 mg/L** |  |  |  |  |  |
| Event proportion | 323/1371 (23.6%) | 320/1215 (26.3%) | 318/1051 (30.3%) | 259/811 (31.9%) |  |
| HR (95%CI) | Ref. | 1.10 (0.94, 1.29) | 1.22 (1.04, 1.43) | 1.21 (1.01, 1.43) | 0.012 |
| **CRP 1-3 mg/L** |  |  |  |  |  |
| Event proportion | 152/599 (25.4%) | 217/670 (32.4%) | 296/844 (35.1%) | 356/943 (37.8%) |  |
| HR (95%CI) | Ref. | 1.24 (1.01, 1.54) | 1.22 (0.99, 1.49) | 1.27 (1.04, 1.56) | 0.053 |
| **CRP ≥ 3 mg/L** |  |  |  |  |  |
| Event proportion | 84/331 (25.4%) | 99/354 (28%) | 141/400 (35.2%) | 231/530 (43.6%) |  |
| HR (95%CI) | Ref. | 1.11 (0.83, 1.5) | 1.40 (1.05, 1.86) | 1.53 (1.17, 2.00) | <0.001 |
| **Heart diseases** | |  |  |  |  |
| **CRP ≤ 1 mg/L** |  |  |  |  |  |
| Event proportion | 274/1371 (20%) | 265/1215 (21.8%) | 262/1051 (24.9%) | 216/811 (26.6%) |  |
| HR (95%CI) | Ref. | 1.05 (0.88, 1.24) | 1.16 (0.97, 1.38) | 1.14 (0.94, 1.38) | 0.085 |
| **CRP 1-3 mg/L** |  |  |  |  |  |
| Event proportion | 129/599 (21.5%) | 172/670 (25.7%) | 245/844 (29.0%) | 282/943 (29.9%) |  |
| HR (95%CI) | Ref. | 1.15 (0.91, 1.45) | 1.17 (0.93, 1.46) | 1.16 (0.93, 1.45) | 0.250 |
| **CRP ≥ 3 mg/L** |  |  |  |  |  |
| Event proportion | 66/331 (19.9%) | 79/354 (22.3%) | 111/400 (27.8%) | 190/530 (35.8%) |  |
| HR (95%CI) | Ref. | 1.12 (0.80, 1.56) | 1.32 (0.96, 1.82) | 1.51 (1.12, 2.05) | 0.003 |
| **Stroke** | |  |  |  |  |
| **CRP ≤ 1 mg/L** |  |  |  |  |  |
| Event proportion | 69/1371 (5%) | 89/1215 (7.3%) | 90/1051 (8.6%) | 76/811 (9.4%) |  |
| HR (95%CI) | Ref. | 1.47 (1.07, 2.02) | 1.61 (1.16, 2.22) | 1.62 (1.15, 2.29) | 0.004 |
| **CRP 1-3 mg/L** |  |  |  |  |  |
| Event proportion | 41/599 (6.8%) | 67/670 (10%) | 92/844 (10.9%) | 135/943 (14.3%) |  |
| HR (95%CI) | Ref. | 1.41 (0.95, 2.11) | 1.37 (0.93, 2.03) | 1.70 (1.17, 2.47) | 0.009 |
| **CRP ≥ 3 mg/L** |  |  |  |  |  |
| Event proportion | 31/331 (9.4%) | 40/354 (11.3%) | 49/400 (12.2%) | 80/530 (15.1%) |  |
| HR (95%CI) | Ref. | 1.25 (0.77, 2.01) | 1.34 (0.84, 2.16) | 1.49 (0.95, 2.34) | 0.082 |

Model adjusted for age, sex, smoking habit, drinking, education level, LDL-C, glucose, SBP, DBP and medication use of lipid-lowering.

**Table S26. Subgroup analysis of changes in CMI with clinical outcomes in different levels of CRP**

|  | **Stable low** | **Low to high** | **High to low** | **Stable high** |
| --- | --- | --- | --- | --- |
| **CVD** | |  |  |  |
| **CRP ≤ 1 mg/L** |  |  |  |  |
| Event proportion | 192/993 (19.3%) | 126/563 (22.4%) | 73/334 (21.9%) | 311/1191 (26.1%) |
| HR (95%CI) | Ref. | 1.16 (0.92, 1.45) | 1.11 (0.84, 1.45) | 1.26 (1.05, 1.52) |
| **CRP 1-3 mg/L** |  |  |  |  |
| Event proportion | 89/436 (20.4%) | 79/279 (28.3%) | 47/205 (22.9%) | 342/1160 (29.5%) |
| HR (95%CI) | Ref. | 1.46 (1.08, 1.99) | 1.17 (0.82, 1.67) | 1.35 (1.06, 1.73) |
| **CRP ≥ 3 mg/L** |  |  |  |  |
| Event proportion | 42/203 (20.7%) | 36/148 (24.3%) | 22/90 (24.4%) | 183/562 (32.6%) |
| HR (95%CI) | Ref. | 1.14 (0.73, 1.80) | 1.11 (0.66, 1.87) | 1.32 (0.93, 1.89) |
| **Heart diseases** | |  |  |  |
| **CRP ≤ 1 mg/L** |  |  |  |  |
| Event proportion | 165/993 (16.6%) | 104/563 (18.5%) | 62/334 (18.6%) | 264/1191 (22.2%) |
| HR (95%CI) | Ref. | 1.08 (0.84, 1.38) | 1.1 (0.82, 1.47) | 1.23 (1.01, 1.50) |
| **CRP 1-3 mg/L** |  |  |  |  |
| Event proportion | 75/436 (17.2%) | 68/279 (24.4%) | 41/205 (20%) | 287/1160 (24.7%) |
| HR (95%CI) |  | 1.47 (1.05, 2.05) | 1.20 (0.82, 1.77) | 1.31 (1.00, 1.71) |
| **CRP ≥ 3 mg/L** |  |  |  |  |
| Event proportion | 31/203 (15.3%) | 33/148 (22.3%) | 21/90 (23.3%) | 155/562 (27.6%) |
| HR (95%CI) | Ref. | 1.46 (0.89, 2.40) | 1.50 (0.86, 2.63) | 1.54 (1.02, 2.31) |
| **Stroke** | |  |  |  |
| **CRP ≤ 1 mg/L** |  |  |  |  |
| Event proportion | 47/993 (4.7%) | 32/563 (5.7%) | 16/334 (4.8%) | 100/1191 (8.4%) |
| HR (95%CI) | Ref. | 1.22 (0.78, 1.92) | 0.97 (0.55, 1.71) | 1.62 (1.13, 2.31) |
| **CRP 1-3 mg/L** |  |  |  |  |
| Event proportion | 20/436 (4.6%) | 22/279 (7.9%) | 10/205 (4.9%) | 125/1160 (10.8%) |
| HR (95%CI) | Ref. | 1.82 (0.98, 3.38) | 1.13 (0.52, 2.44) | 2.22 (1.35, 3.66) |
| **CRP ≥ 3 mg/L** |  |  |  |  |
| Event proportion | 18/203 (8.9%) | 11/148 (7.4%) | 8/90 (8.9%) | 62/562 (11.0%) |
| HR (95%CI) | Ref. | 0.88 (0.41, 1.88) | 0.97 (0.42, 2.26) | 1.11 (0.63, 1.94) |

Model adjusted for age, sex, smoking habit, drinking, education level, LDL-C, glucose, SBP, DBP and medication use of lipid-lowering.

**Table S27. Subgroup analysis of cumulative CMI with CVD in different levels of CRP**

|  | **T1** | **T2** | **T3** | **P for trend** |
| --- | --- | --- | --- | --- |
| **Participants with low baseline CMI** | |  |  |  |
| **CRP ≤ 1 mg/L** |  |  |  |  |
| Event proportion | 32/154 (20.8%) | 154/804 (19.2%) | 132/598 (22.1%) |  |
| HR (95%CI) | Ref. | 0.84 (0.57, 1.23) | 1.01 (0.68, 1.50) | 0.402 |
| **CRP 1-3 mg/L** |  |  |  |  |
| Event proportion | 9/56 (16.1%) | 84/375 (22.4%) | 75/284 (26.4%) |  |
| HR (95%CI) | Ref. | 1.68 (0.83, 3.37) | 2.01 (0.99, 4.07) | 0.048 |
| **CRP ≥ 3 mg/L** |  |  |  |  |
| Event proportion | 7/41 (17.1%) | 33/151 (21.9%) | 38/159 (23.9%) |  |
| HR (95%CI) | Ref. | 0.94 (0.41, 2.15) | 1.12 (0.49, 2.55) | 0.575 |
| **Participants with high baseline CMI** | |  |  |  |
| **CRP ≤ 1 mg/L** |  |  |  |  |
| Event proportion | 193/799 (24.2%) | 76/306 (24.8%) | 115/420 (27.4%) |  |
| HR (95%CI) | Ref. | 1.08 (0.82, 1.41) | 1.12 (0.88, 1.42) | 0.334 |
| **CRP 1-3 mg/L** |  |  |  |  |
| Event proportion | 206/686 (30.0%) | 85/273 (31.1%) | 98/406 (24.1%) |  |
| HR (95%CI) | Ref. | 1.03 (0.80, 1.34) | 0.77 (0.61, 0.99) | 0.056 |
| **CRP ≥ 3 mg/L** |  |  |  |  |
| Event proportion | 107/340 (31.5%) | 36/131 (27.5%) | 62/181 (34.3%) |  |
| HR (95%CI) | Ref. | 0.91 (0.62, 1.34) | 1.08 (0.78, 1.50) | 0.704 |

Model adjusted for age, sex, smoking habit, drinking, education level, LDL-C, glucose, SBP, DBP and medication use of lipid-lowering.

**Table S28. Subgroup analysis of ΔCMI with CVD in different levels of CRP**

|  | **ΔT1** | **ΔT2** | **ΔT3** | **P for trend** |
| --- | --- | --- | --- | --- |
| **Participants with low baseline CMI** | |  |  |  |
| **CRP ≤ 1 mg/L** |  |  |  |  |
| Event proportion | 132/598 (22.1%) | 32/154 (20.8%) | 154/804 (19.2%) |  |
| HR (95%CI) | 0.99 (0.67, 1.46) | 0.83 (0.66, 1.05) | Ref. | 0.117 |
| **CRP 1-3 mg/L** |  |  |  |  |
| Event proportion | 75/284 (26.4%) | 9/56 (16.1%) | 84/375 (22.4%) |  |
| HR (95%CI) | 0.50 (0.25, 1.01) | 0.83 (0.61, 1.15) | Ref. | 0.301 |
| **CRP ≥ 3 mg/L** |  |  |  |  |
| Event proportion | 38/159 (23.9%) | 7/41 (17.1%) | 33/151 (21.9%) |  |
| HR (95%CI) | 0.89 (0.39, 2.04) | 0.84 (0.51, 1.37) | Ref. | 0.479 |
| **Participants with high baseline CMI** | |  |  |  |
| **CRP ≤ 1 mg/L** |  |  |  |  |
| Event proportion | 115/420 (27.4%) | 193/799 (24.2%) | 76/306 (24.8%) |  |
| HR (95%CI) | 0.89 (0.70, 1.13) | 0.96 (0.72, 1.29) | Ref. | 0.711 |
| **CRP 1-3 mg/L** |  |  |  |  |
| Event proportion | 98/406 (24.1%) | 206/686 (30%) | 85/273 (31.1%) |  |
| HR (95%CI) | 1.29 (1.01, 1.65) | 1.34 (1.00, 1.79) | Ref. | 0.040 |
| **CRP ≥ 3 mg/L** |  |  |  |  |
| Event proportion | 62/181 (34.3%) | 107/340 (31.5%) | 36/131 (27.5%) |  |
| HR (95%CI) | 0.93 (0.67, 1.29) | 0.84 (0.56, 1.27) | Ref. | 0.410 |

Model adjusted for age, sex, smoking habit, drinking, education level, LDL-C, glucose, SBP, DBP and medication use of lipid-lowering.

**Table S29. Changes of factors associated with CVD during follow-up in the participants with high baseline CMI level***

| **Factors** | **High to low**  **(n = 629)** | **Stable high**  **(n = 2913)** | ***P value*** |
| --- | --- | --- | --- |
| Quit smoking, n (%) | 34.0 (5.4%) | 147.0 (5.0%) | 0.8 |
| Quit drinking, n (%) | 36.0 (5.7%) | 151.0 (5.2%) | 0.7 |
| Return to normal BMI, n (%) | 65.0 (10.4%) | 191.0 (6.6%) | 0.001 |
| Increase in physical activity, n (%) | 86.0 (40.8%) | 308.0 (29.0%) | 0.001 |
| Return to normal sleep, n (%) | 97.0 (15.8%) | 353.0 (12.4%) | 0.027 |
| Start Nap, n (% | 89.0 (14.4%) | 469.0 (16.4%) | 0.3 |
| Increase in hsCRP, n (%) | 98.0 (15.6%) | 792.0 (27.2%) | <0.001 |
| Start lipid-lowering drugs, n (%) | 26.0 (4.1%) | 220.0 (7.6%) | 0.003 |
| New diabetic diagnostic, n (%) | 55.0 (8.7%) | 415.0 (14.2%) | <0.001 |
| New hypertension diagnostic, n (%) | 69.0 (11.0%) | 317.0 (10.9%) | >0.9 |

* Only the participants with measurements at both baseline and follow-up (Wave 3) were included.

Definitions: **Quit smoking** - current smoker at baseline who quit at Wave 3. **Quit drinking** **-**current drinker at baseline who quit at Wave 3. **Return to normal BMI** – baseline BMI<18.5 or ≥24, and BMI 18.5~24 at Wave 3. **Increase in physical activity** **–** mild or walking at baseline and moderate-to-vigorous at Wave 3. **Return to normal sleep** - sleep <6 hours/night at baseline, and ≥6 hours/night at Wave 3. **Start Nap** - no nap at baseline, and nap taken at Wave 3. **Increase in hsCRP** - hsCRP <2 mg/L at baseline, and hsCRP ≥2 mg/L at Wave 3. **New diabetes/hypertension diagnosis** – newly diagnosed between baseline and Wave 3.

**Table S30. Incremental value of adding changes in CMI to baseline model**

|  | **Value/slope** | **95%CI** |
| --- | --- | --- |
| **Δ C-statistic** | 0.007 | (0.003, 0.012) |
| **IDI** | 0.002 | (0.0007, 0.0035) |
| **Continuous NRI** | 0.274 | (0.135, 0.364) |
| **Category-free NRI** | 0.018 | (-0.018, 0.051) |
| **Calibration slope-base model** | 0.841 | (0.630, 1.050) |
| **Calibration slope-extended model** | 0.906 | (0.700, 1.110) |

IDI: integrated discrimination improvement;

Continuous NRI: category-free net reclassification improvement;

Category-based NRI: calculated using predefined risk categories (<5%, 5-10% and ≥10%).

Base model including baseline CMI, age, sex, smoking habit, drinking, education level, LDL-C, glucose, SBP, DBP and medication use of lipid-lowering.

Extended model including baseline CMI, age, sex, smoking habit, drinking, education level, LDL-C, glucose, SBP, DBP, medication use of lipid-lowering and changes in CMI.

**Figure S4. Decile-based calibration plots at 3-year.**

**Table S31. Fine-Gray competing-risks regression for cardiovascular mortality**

| **Variable** | **SHR** | **95%CI** | **P value** |
| --- | --- | --- | --- |
| Quartiles of baseline CMI |  |  |  |
| Q2 vs Q1 | 1.15 | 1.03–1.29 | 0.012 |
| Q3 vs Q1 | 1.27 | 1.14–1.42 | <0.001 |
| Q4 vs Q1 | 1.34 | 1.20–1.49 | <0.001 |
| Age (per year) | 1.03 | 1.02–1.03 | <0.001 |
| Male sex | 1.13 | 1.02–1.25 | 0.015 |
| Smoking* | 0.92 | 0.83–1.02 | 0.104 |
| Drinking | 0.98 | 0.90–1.07 | 0.646 |
| Higher education* | 1.43 | 1.06–1.92 | 0.018 |
| Lipid-lowering medication | 1.92 | 1.73–2.14 | <0.001 |
| LDL-C | 1.04 | 1.00–1.08 | 0.066 |
| SBP | 1.00 | 1.00–1.01 | 0.003 |
| DBP | 1.01 | 1.00–1.01 | <0.001 |
| Glucose | 1.01 | 1.00–1.03 | 0.131 |
| CRP | 1.00 | 1.00–1.01 | 0.574 |

*the “never” and “former” categories of the smoking habits variable was combined into one; the “low” and “Middle” of education level variable was combined into one.

SHR: sub-distribution hazard ration. Cardiovascular disease was treated as the event of interest, and non-cardiovascular death was treated as a competing event.

**Figure S5. The cumulative incidence of CVD according to quartiles of baseline CMI, accounting for non-cardiovascular death as a competing event.**

**Reference**

1.	Chen X, Crimmins E, Hu P, et al. Venous Blood-based Biomarkers in the China Health and Retirement Longitudinal Study: Rationale, Design, and Results of the 2015 Wave. *American Journal of Epidemiology.* 2019;188(11):1871–1877.

2.	Zhao Y, Hu Y, Smith JP, Strauss J, Yang G. Cohort Profile: The China Health and Retirement Longitudinal Study (CHARLS). *International Journal of Epidemiology.* 2012;43(1):61-68.

3.	He D, Wang Z, Li J, et al. Changes in frailty and incident cardiovascular disease in three prospective cohorts. *European Heart Journal.* 2024;45(12):1058-1068.

4.	White IR, Royston P, Wood AM. Multiple imputation using chained equations: Issues and guidance for practice. *Statistics in medicine.* 2011;30(4):377-399.
